# Recomb-Mix: fast and accurate local ancestry inference

**DOI:** 10.1093/bioinformatics/btaf227

**Published:** 2025-07-15

**Authors:** Yuan Wei, Degui Zhi, Shaojie Zhang

**Affiliations:** Department of Computer Science, University of Central Florida, Orlando, FL 32816, United States; McWilliams School of Biomedical Informatics, University of Texas Health Science Center at Houston, Houston, TX 77030, United States; Department of Computer Science, University of Central Florida, Orlando, FL 32816, United States

## Abstract

**Motivation:**

The availability of large genotyped cohorts brings new opportunities for revealing the high-resolution genetic structure of admixed populations via local ancestry inference (LAI), the process of identifying the ancestry of each segment of an individual haplotype. Though current methods achieve high accuracy in standard cases, LAI is still challenging when reference populations are more similar (e.g. intra-continental), when the number of reference populations is too numerous, or when the admixture events are deep in time, all of which are increasingly unavoidable in large biobanks.

**Results:**

In this work, we present Recomb-Mix, a new LAI method which integrates elements from the site-based Li and Stephens model and introduces a new graph collapsing techniques to simplify counting paths with the same ancestry label readout. Through comprehensive benchmarking on various simulated datasets, we show that Recomb-Mix is more accurate than existing methods in diverse sets of scenarios while being competitive in terms of resource efficiency. The scalability and robustness of Recomb-Mix are also demonstrated with real-world datasets. We expect that Recomb-Mix will be a useful method for advancing genetics studies of admixed populations.

**Availability and implementation:**

The implementation of Recomb-Mix is available at https://github.com/ucfcbb/Recomb-Mix.

## 1 Introduction

Local ancestry inference (LAI) is a process of assigning the ancestral population labels of each segment on an individual’s genome sequence. LAI is not only useful for better study of human demographic history (Martin *et al.* 2017) but also can enable several downstream tasks, including admixture mapping (Reich *et al.* 2005), ancestry-aware genome-wide association studies (GWAS) (Pasaniuc *et al.* 2011), and ancestry-specific polygenic risk scores (Duncan *et al.* 2019). Recent studies show that local ancestry information improves the resolution of association signals in GWAS (Atkinson *et al.* 2021), helping to infer the high-resolution of genomic regions containing genes as under selection (Hamid *et al.* 2023). Local ancestry calls contribute to understanding the impact of genetic variants that cause disease (Hou *et al.* 2023) and the accuracy of polygenic scores of genetically based predictions (Ding *et al.* 2023).

The recent availability of genotyped datasets on a biobank scale (Bycroft *et al.* 2018, Kurki *et al.* 2023) and the rising of enormous databases from direct-to-consumer genetic companies (Durand *et al.* 2021, Wang *et al.* 2021) create new challenges and opportunities for LAI. Participants in biobanks may be from highly imbalanced source populations. Inferring ancestral components underrepresented in these reference populations is in great need. On the other hand, the admixture to be inferred in the samples may be multi-ways and may be from both recent and distant admixture events. However, opportunities coexist with such challenges. More diverse samples, e.g. the Human Genome Diversity Project (HGDP) (Bergström *et al.* 2020), are becoming available as reference panels. The number of participants in biobanks is much larger and more representative than in previous reference panels, and thus, more potential sub-continental ancestral information becomes available from biobanks. Although methods for revealing sub-continental or even sub-population clusters are available (e.g. Lawson *et al.* 2012), they are mostly non-LAI methods and only capture global ancestry. With the availability of diverse samples in biobanks and the need for in-depth knowledge of admixed individuals, current LAI methods are unable to capitalize on these opportunities fully.

Existing LAI methods fall into two categories: site-based and window-based. Originally, HapMix (Price *et al.* 2009), based on the extended Li and Stephens (LS) Hidden Markov model (HMM) (Li and Stephens 2003), is developed to model different transition probabilities for within population and between population jumps. To make it tolerate mismatches, emission probabilities are also introduced, and the model is not very efficient and cannot be scaled up (Geza *et al.* 2019). Later, window-based methods gained popularity [e.g. RFMix (Maples *et al.* 2013), G-Nomix (Hilmarsson *et al.* 2021), SALAI-Net (Oriol Sabat *et al.* 2022)]. These methods take short stretches of sites as windows and define window-based features. They first make local ancestry prediction over each window and then apply post-processing techniques to smooth the labels across all windows. Typically, a specific machine-learning approach is used for each window. However, the predefined window boundaries are not necessarily optimized, and the noisy initial window labels can be difficult to correct by post-processing. Loter (Dias-Alves *et al.* 2018) is a recent site-based LAI method. It formulates the LAI problem as a combinatorial best-path problem in a graph, which can be solved efficiently by dynamic programming. However, its problem formulation is simplistic in that it does not take into account the useful information encoded in the LS model, such as differential transition probabilities for within and between populations and variable recombination rates across sites. Loter reported that it underperformed RFMix (Maples *et al.* 2013) and LAMP-LD (Baran *et al.* 2012) for datasets with recent admixture events (i.e. <150 generations). Therefore, there is room for improvement over the Loter approach by introducing an LS-inspired parametrization of its scoring function.

In this work, we developed Recomb-Mix, a site-based method that is both accurate and efficient. Our main insight is that we do not have to have an exact LS formulation. The HapMix LS model incorporates differential transition penalties for within and between populations, assigning the population labels to a site by comparing paths going through it versus paths by-passing it. We achieve a similar result by setting the within-population transition penalty to zero and collapsing the nodes representing the allele values at each site. This allows both run time and space efficiency while achieving higher accuracy than Loter. Recomb-Mix is designed with the goals of robustness, scalability, and superior accuracy on LAI. Applications to real human datasets confirmed the genetic differences among populations and provided potential explanations for how the evolutionary processes shape these differences.

## 2 Materials and methods

### 2.1 Recomb-Mix

The Recomb-Mix method is inspired by the LS HMM and follows Loter which frames the ancestry prediction problem as a graph optimization problem. It assumes that an admixed individual haplotype is modeled as a mosaic of individual haplotypes from a reference panel. Recomb-Mix constructs a population graph from a given reference panel to infer the ancestral label at each locus on a given admixed individual haplotype by finding a threading path that resembles the admixed individual haplotype the most among all the paths. In the population graph, the allele values of individual haplotypes are grouped by each site. Then, the population graph is transformed into a compact population graph by collapsing the site nodes with the same allele value and ancestral label into one node. A mismatch penalty at each site occurs when there is a difference between the collapsed site nodes’ allele value and the corresponding site’s allele value in the admixed individual haplotype. A template change penalty regarding recombinations between each site occurs when two nodes of adjacent sites having different ancestral labels are connected. This process is similar to the HMM Forward algorithm (Durbin *et al.* 1998) as each node in the compact population graph can be viewed as a bundle of nodes in the original population graph being consolidated as one, and their ancestral labels are assigned by all low penalty threading paths passing through it. Recomb-Mix sums over mismatch penalties and template change penalties through all possible threading paths to determine the one that has the minimum penalty score. The ancestral label of each site in the admixed individual haplotype is assigned the same ancestral label of each corresponding node on such a threading path.

**Figure 1.**
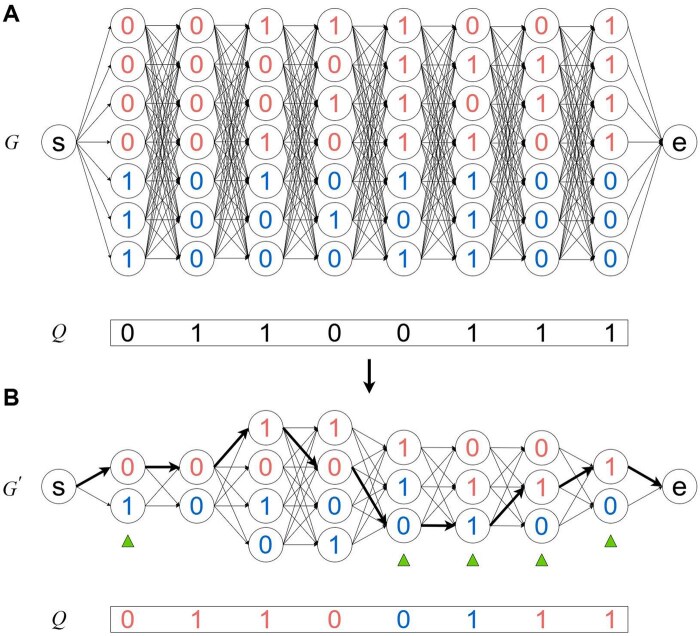
An example of local ancestry inference with Recomb-Mix. (A) *G* is a population graph constructed from a given reference panel. *G* contains seven haplotypes with eight sites belonging to two populations (shown in red and blue). *Q* is a query of an admixed individual haplotype. (B) $G^{\prime }$ is a compact population graph transformed from *G* by collapsing the nodes having the same allele value and ancestral label per site. Sites marked with green triangles are dAIMs. *Q* is assigned with estimated ancestral labels for each site (shown in red and blue on allele values), according to a threading path selected with minimum penalty score (shown as bold edges) in $G^{\prime }$.

Figure 1 is an example of local ancestry inference with Recomb-Mix. A reference panel having seven individual haplotypes, eight sites, and two ancestral labels (shown in red and blue) is represented as a population graph *G*. Nodes representing each site are fully connected to nodes representing their adjacent site. A node *s* is connected to all nodes for the first site, and all nodes for the last site are connected to a node *e*. *Q* is a query of an admixed individual haplotype. *G* is then transformed into a compact population graph $G^{\prime }$ by collapsing nodes with the same allele value and ancestral label to one node. The penalty score of each path is calculated based on the nodes (i.e. site allele values between $G^{\prime }$ and *Q*), and the edges (i.e. path penalties when the connected nodes’ ancestral labels differ) in $G^{\prime }$. Finally, a threading path having the minimum penalty score is selected from node *s* to node *e* in $G^{\prime }$, to be used to paint the admixed individual haplotype query by assigning the estimated ancestral label to each site in *Q*.

Local ancestry inference may benefit from ancestry informative markers (AIMs), which are genetic markers with significantly different allele frequencies in various populations (Parra *et al.* 1998). AIMs provide information regarding ancestry and can be determined in a panel by measuring marker informativeness for ancestry (Ding *et al.* 2011). Rather than relying on a selected set of markers, i.e. AIMs, the proposed compact population graph keeps all markers, and the only differential information between populations is that some alleles might be missing in one or several populations. These markers are referred to as discrete AIMs (dAIMs), which have allele values present in the panel of one population but absent in at least one other population. Recomb-Mix’s power of discriminating different populations comes from the existence of dAIMs that show different sets of alleles in different populations after collapsing, e.g. markers with green triangles in $G^{\prime }$ in Fig. 1.

As a site-based LAI method, Recomb-Mix shares many common elements with other site-based methods (Supplementary Table S1). However, Recomb-Mix utilizes a new parameterization and scoring function designed to achieve graph simplification and ancestry signal amplification. Two key insights of Recomb-Mix make it better at capturing ancestry signals. First, it focuses on population-specific allele values from a compressed reference panel rather than on allele frequencies. Second, it does not impose the template change penalty when the path switches within the same population, thereby simplifying the algorithmic complexity while amplifying ancestry signals.

### 2.2 Graph optimization formulation

To formally define the Recomb-Mix method, a reference panel having *m* individual haplotypes with *n* sites can be transformed as a population graph G=(V,E), representing this set of haplotypes. *V* is the union of the starting and the ending nodes {s,e} and the site nodes $S_{j}$ for j∈[1,n]. $S_{j}=\{s_{j}^{1},s_{j}^{2}\ldots s_{j}^{m}\}$ is the set of nodes representing alleles of haplotypes at position *j*. *E* is the union of the edges from $s_{j}^{i}$ to $s_{j+1}^{k}$ for all j∈[1,n−1], i,k∈[1,m], and the edges from *s* to $s_{1}^{i}$ and $s_{n}^{i}$ to *e* for i∈[1,m]. $s_{j}^{i}$ is the node that the *i*th haplotype has at site *j*. Each node at every site of every haplotype has an associated ancestral label. It is assumed there are *p* populations presented in the reference panel, and each population has an ancestral label in *[*1, *p]*. The ancestral label of node $s_{j}^{i}$ is $l(s_{j}^{i})\in [1,p]$. The allele value of node $s_{j}^{i}$ is $a(s_{j}^{i})\in [0,1]$, assuming all sites are bi-allelic. It can be easily extended to the case with multi-allelic values: for K alleles, $a(s_{j}^{i})\in [0,K-1]$. The population graph *G* is further transformed into a compact population graph $G^{\prime }=(V^{\prime },E^{\prime })$ by collapsing all nodes with the same allele value and ancestral label to one node in every site. $V^{\prime }$ is the union of the starting and the ending nodes {s,e} and the site nodes $S^{\prime }{}_{j}=\{s^{\prime }{}_{j}^{1},s^{\prime }{}_{j}^{2},\ldots ,s^{\prime }{}_{j}^{\left|S^{\prime }{}_{j}\right|}\}$ for j∈[1,n]. $S^{\prime }{}_{j}$ is a set of nodes representing all unique pairs of allele values and ancestral labels in $S_{j}$ (i.e. there is a node $s^{\prime }{}_{j}^{i}\in S^{\prime }{}_{j}$ if and only if there is a node $s\in S_{j}$ such that $a(s)=a(s^{\prime }{}_{j}^{i})$ and $l(s)=l(s^{\prime }{}_{j}^{i})$ and for all $k\in [1,|S^{\prime }{}_{j}|],k\neq i$, $a(s^{\prime }{}_{j}^{i})\neq a(s^{\prime }{}_{j}^{k})$ or $l(s^{\prime }{}_{j}^{i})\neq l(s^{\prime }{}_{j}^{k})$). $E^{\prime }$ is the union of the edges from $u_{1}$ to $u_{2}$ for all $u_{1}\in S^{\prime }{}_{j}$ and $u_{2}\in S^{\prime }{}_{j+1}$ for j∈[1,n−1], and the edges from *s* to $u_{1}$ and $u_{2}$ to *e* for all $u_{1}\in S^{\prime }{}_{1}$ and $u_{2}\in S^{\prime }{}_{n}$.

To calculate all possible threading paths in $G^{\prime }$ for a query admixed individual haplotype *Q* with *n* sites, $Q=(q_{1},q_{2},\ldots q_{n})$ (the allele value of $q_{i}$ is $a(q_{i})\in [0,1]$), Recomb-Mix incorporates the mismatch penalty and the template change penalty into its objective function. The mismatch penalty function is defined as $d(x_{1},x_{2})$, where $x_{1}$ and $x_{2}$ are allele values. The template change penalty function is defined as $r(y_{1},y_{2})$, where $y_{1}$ and $y_{2}$ are ancestral label values. Then, the cost of a candidate threading path $P=(u_{1},u_{2},\ldots ,u_{n})$ is defined as:


(1) $$f(P)=\sum_{j=1}^{n}d(a(q_{j}),a(u_{j}))+w\sum_{j=1}^{n-1}r(l(u_{j}),l(u_{j+1})).$$

In Equation (1), *w* is a scale factor to balance the mismatch and recombination costs. Let $P^{*}$ be the threading path having the minimum penalty cost among all candidate threading paths in $G^{\prime }$. The ancestral labels of the nodes in $P^{*}$ from *s* to *e* are the estimated ancestral labels of sites in *Q*, that is $\left(l(u^{*}{}_{1}),l(u^{*}{}_{2}),\ldots ,l(u^{*}{}_{n})\right)$. Thus, LAI can be formulated as a problem to find $P^{*}$ in $G^{\prime }$.

Recomb-Mix uses a simplified scoring function [Equation (1)] like the one HapMix uses to calculate the penalty score of each threading path. The mismatch penalty is determined by simply comparing the allele values of each site in *Q* to the corresponding nodes in $G^{\prime }$. For each site, a mismatch penalty is applied if the allele values are not the same. d(·) in Equation (1) is implemented as $d(a(q_{j}),a(u_{j}))=0$ if $a(q_{j})=a(u_{j})$; otherwise $d(a(q_{j}),a(u_{j}))=1$ for site *j* of $u_{j}$ in $G^{\prime }$ and $q_{j}$ in *Q*. The template change penalty is determined by the recombination rate between site *j* and j+1 and the ancestral labels of $u_{j}$ and $u_{j+1}$ in $G^{\prime }$. r(·) in Equation (1) is implemented as $r(l(u_{j}),l(u_{j+1}))=0$ if $l(u_{j})=l(u_{j+1})$; otherwise $r(l(u_{j}),l(u_{j+1}))=R_{j,j+1}$ for site *j* and j+1 of $u_{j}$ and $u_{j+1}$ in $G^{\prime }$. $R_{j,j+1}$ is the normalized reciprocal of the recombination rate between site *j* and j+1. To calculate $R_{j,j+1}$, the min-max normalization method (Kotsiantis *et al.* 2006) is used to scale the range of the recombination rates from the genetic map into [0, 1], where the recombination rate is calculated as the genetic distance in centiMorgan over the physical distance in Megabase between sites. The normalized recombination rates $R_{j,j+1}$ are further processed by applying a reciprocal function to obtain $R_{j,j+1}$, where $R_{j,j+1}=2/(R_{j,j+1}+1)$. The range of the normalized reciprocal of the recombination rates is [1, 2]. The goal of having the normalization step and the scale factor is to ensure the template change penalty is in the same order of magnitude as the mismatch penalty to prevent the domination of any penalties.

Representing the LS model as a compact population graph is efficient in terms of time and space. Recomb-Mix uses a dynamic programming approach to solve the problem of finding $P^{*}$ in $G^{\prime }$ (see Supplementary Algorithm). Using $G^{\prime }$ instead of *G* to compute the minimum penalty score among all possible threading paths significantly reduces the computing time and the demand for spaces to store the reference panel. The maximum indegree and outdegree of any node in $G^{\prime }$ is a constant value 2*p*, assuming all sites are bi-allelic and the number of populations presented in the reference panel is small (i.e. p≪m). Therefore, both the time and space complexity of computing the penalty scores on $G^{\prime }$ are O(np).

Another advantage of presenting a reference panel as a compact population graph is the panel does not have to be a phased one. When converting a reference panel into a compact population graph, the order of the sites from two haplotypes of an individual is irrelevant, thanks to a property that the out-neighborhood of a node *u* in a graph is the set of nodes adjacent to *u*. Thus, Recomb-Mix is flexible to handle both phased and unphased reference panels.

### 2.3 Simulated dataset

To evaluate the performance of Recomb-Mix, several admixture datasets were simulated using SLiM v4.0 (Haller and Messer 2019). The input data were individuals in Whole-Genome Sequencing (WGS) form from various populations in the study of the 1000 Genomes Project (TGP) (Auton *et al.* 2015, Clarke *et al.* 2017) and the Human Genome Diversity Project (HGDP) (Bergström *et al.* 2020). The founder and reference individuals were separated as independent populations at the beginning of the simulation. The admixture population was simulated as the descendants of admixed founders from different populations. The admixed individuals were sampled from the admixture population, and the referenced individuals were sampled from each reference population. A set of three-way inter-continental datasets of Chromosome 18 were simulated using YRI, CEU, and CHB individuals, representing African, European, and Asian populations from the TGP dataset. Detailed descriptions of the populations are available in Supplementary Table S2. Various sizes of reference panels (i.e. 100, 250, 500, and 1000) and numbers of generations after the admixture event (i.e. 15, 50, 100, and 200) were examined. The average recombination rate and mutation rate used for the simulation were $1.46\times 10^{-8}$ and $1.29\times 10^{-8}$ per base pair per generation, according to the stdpopsim library (Adrion *et al.* 2020). A 0.02% genotyping error, following Browning *et al.* (Browning *et al.* 2023), was added to admixed and reference individuals. The ground truth ancestral labels of admixed individuals were extracted from the SLiM output tree sequence (Haller *et al.* 2019). Additionally, a set of seven-way inter-continental datasets of Chromosome 18 were simulated using AFR, EAS, EUR, NAT, OCE, SAS, and WAS individuals (representing African, East Asian, European, American, Oceanian, Central and South Asian, and West Asian populations) from the HGDP dataset. The HGDP dataset was phased and imputed using Beagle 5.4 (Browning *et al.* 2018, 2021). The goal of simulating the seven-way admixture is to explore how well LAI methods are able to distinguish local ancestral segments from the admixture of a large number of ancestral populations.

To explore the power of LAI at the intra-continental level, a set of intra-continental datasets was simulated using TSI, FIN, and GBR individuals (representing Italian, Finnish, and British populations) using the same settings and population sizes as the inter-continental datasets. To explore the influence of the uneven proportion of individuals per population in founders and references, two variations of the three-way 15-generation intra-continental datasets with uneven founders (i.e. 68 Italian, 32 Finnish, and 100 British individuals) or uneven references (i.e. 170 Italian, 80 Finnish, and 250 British individuals) were simulated. Both cases were 1/3, 1/6, and 1/2 individuals to the entire population panel. Additionally, an experiment was conducted to test the case when the reference panel size was ultra-small, i.e. the reference panel size was 20 and 50 (or only about 7 or 17 individuals per population in the reference panel).

### 2.4 Benchmark setup

Two conventional measurements were used to evaluate the performance of LAI methods. The squared Pearson’s correlation coefficient $r^{2}$ value [used by FLARE, LAMP-LD (Baran *et al.* 2012), and MOSAIC (Salter-Townshend and Myers 2019)] and the accuracy rate of the correctly predicted markers (used by G-Nomix, Loter, RFMix, and SALAI-Net). The $r^{2}$ value was followed by LAMP-LD’s definition (Baran *et al.* 2012), in which the $r^{2}$ value is defined as the one between the true and the inferred number of alleles from each of the populations, averaged over all the populations. The accuracy rate is defined as the number of correctly inferred markers over the total number of markers. The criteria used by FLARE that markers were filtered with minor allele frequency ≤ 0.005 and minor allele count ≤ 50 (Browning *et al.* 2023) was also applied. $r^{2}$ values are mainly reported in the benchmarks but accuracy rates are also available, mostly in the Supplementary Materials. It is found that the results of $r^{2}$ values and accuracy rates are often consistent.

Recomb-Mix was tested with several datasets against the following LAI methods: FLARE (Browning *et al.* 2023), G-Nomix (Hilmarsson *et al.* 2021), Loter (Dias-Alves *et al.* 2018), RFMix (Maples *et al.* 2013), and SALAI-Net (Oriol Sabat *et al.* 2022). The weight parameter *w* was tuned to w=1.5, which provides the best performance of Recomb-Mix for the given datasets. Parameters used for other methods are available in Supplementary Table S3. FLARE, a site-based generative method extended from HapMix (Price *et al.* 2009), models the hidden local ancestry of each site. It is designed to be flexible for providing optional parameters to deal with various situations. G-Nomix is a window-based discriminative approach using two-stage prediction to perform LAI, in which it uses Logistic Regression as a base model and XGBoost as a smoother model. Loter frames the ancestry prediction problem as a graph optimization problem. It is prioritized for LAI on distant admixture events and is good for non-model species as no biological information is required. RFMix, a window-based discriminative approach, applies a conditional random field model to LAI, particularly using random forest classification. SALAI-Net, a window-based discriminative approach, uses a reference matching layer and a smoother layer (i.e. a combination of cosine similarity score and neural network) to perform LAI, with the adoption of GPU hardware and pre-trained generalized models.

## 3 Results

### 3.1 Local ancestry inference for three-way inter-continental admixed populations

**Figure 2.**
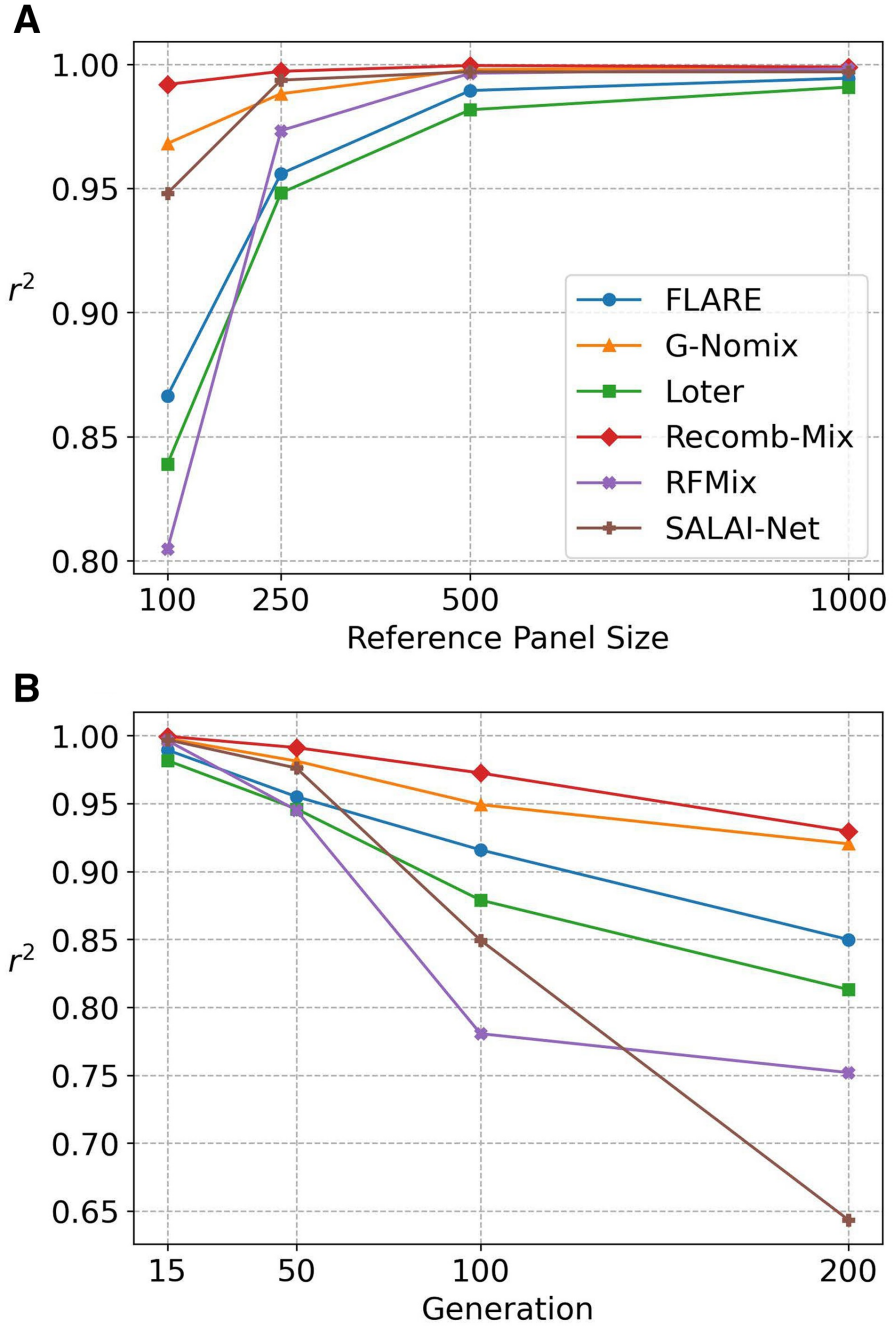
The squared Pearson’s correlation coefficient $r^{2}$ of three-way inter-continental simulated datasets on FLARE, G-Nomix, Loter, Recomb-Mix, RFMix, and SALAI-Net. Markers were filtered with minor allele frequency ≤0.005 and minor allele count ≤50. (A) The three-way 15-generation datasets with the reference panel sizes 100, 250, 500, and 1000 (values are in Supplementary Table S4). (B) The three-way 500-reference datasets with the generations 15, 50, 100, and 200 (values are in Supplementary Table S5).

For three-way inter-continental simulated datasets, Recomb-Mix had the best $r^{2}$ values and accuracy rates in reference panel sizes 100, 250, 500, and 1000 with 15 generations and in generations 15, 50, 100, and 200 with 500 references. Figure 2 shows the $r^{2}$ values of the inference results on six LAI methods, FLARE, G-Nomix, Loter, Recomb-Mix, RFMix, and SALAI-Net. The accuracy rates are shown in Supplementary Figs S1 and S2 (values are in Supplementary Tables S6 and S7). Overall, as the reference panel size increases, the average $r^{2}$ value and the accuracy rate increase for all methods. The large reference panel containing more individual samples than those in small ones helps improve the inference result. When using a reference panel with 1000 individuals, all methods had at least 0.99 $r^{2}$ value or 92% accuracy rate, while Recomb-Mix reached the best $r^{2}$ value 0.9989 or accuracy rate of 99.10%. Recomb-Mix performed well for a small reference panel (i.e. for a 100-individual penal it achieved the best performance, that is $r^{2}$ value 0.9919 or accuracy rate of 97.96%). G-Nomix and SALAI-Net had the second and the third highest $r^{2}$ values, which were 0.9681 and 0.9480. SALAI-Net and G-Nomix had the second and the third highest accuracy rates, which were 86.69% and 86.63%.

Recomb-Mix’s performance on ultra-small reference panels (i.e. size 20–50) was tested. Such cases are interesting because small reference panels can benefit low-resourced populations. Meanwhile, LAI with small reference panels is challenging because allele frequencies and haplotype frequencies are noisy. For a small reference panel size of 20, Recomb-Mix achieves the best accuracy rate of 62.85%; for a 50-individual reference panel, Recomb-Mix achieves 94.45%, while other methods’ accuracy rates are around 60%–70%. See Supplementary Fig. S3 and Supplementary Table S8. We include MOSAIC (Salter-Townshend and Myers 2019) in this experiment as it reportedly performs well on small reference panels (Browning *et al.* 2023). MOSAIC achieves better accuracy rates than FLARE, Loter, and RFMix on reference panels of sizes 50 and 100, but its performance is worse than Recomb-Mix.

### 3.2 Multi-way admixture

**Figure 3.**
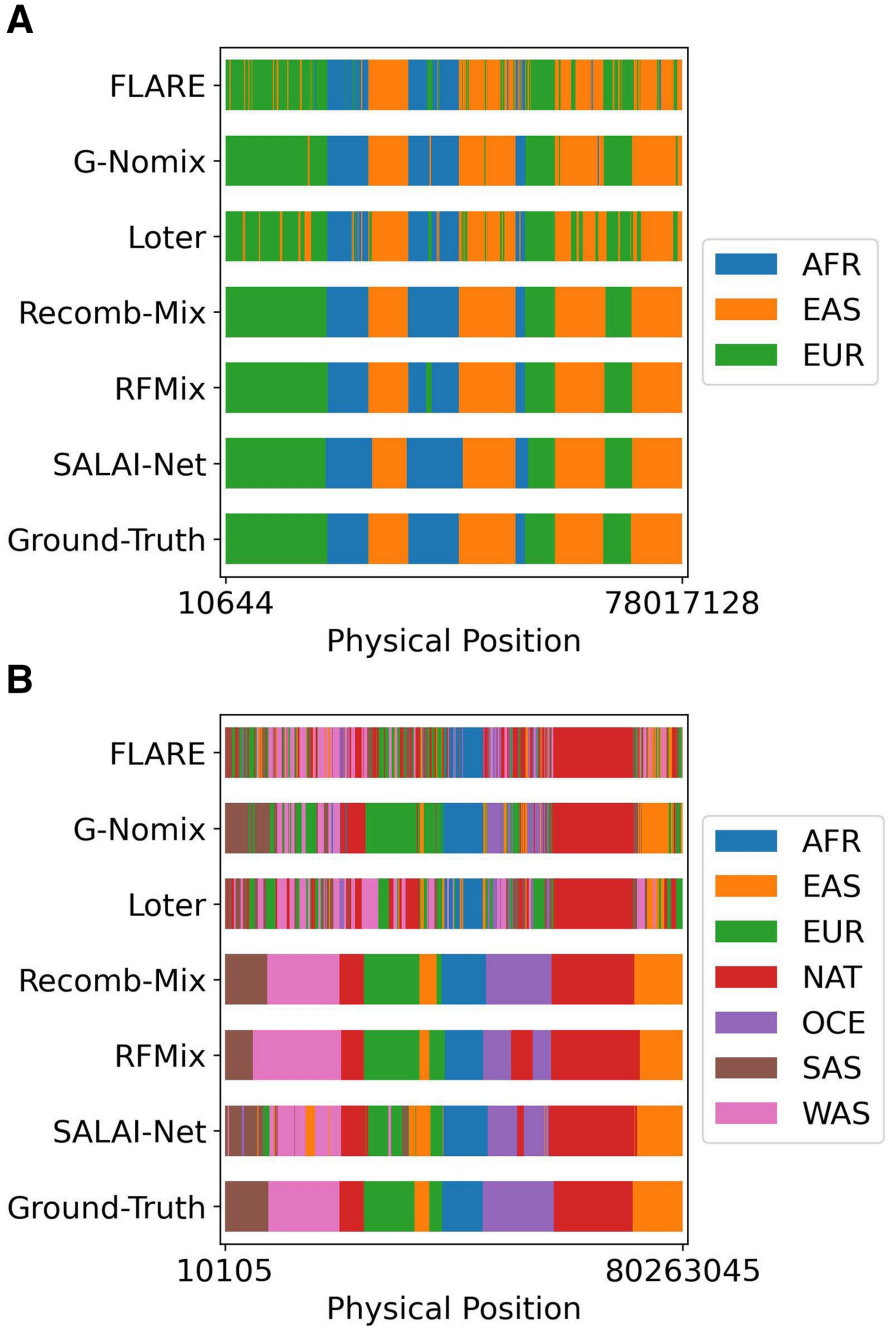
Sample haplotypes inferred by FLARE, G-Nomix, Loter, Recomb-Mix, RFMix, and SALAI-Net with the ground truth of ancestry labels. (A) An inferred sample haplotype from a three-way 15-generation 500-reference inter-continental simulated dataset. (B) An inferred sample haplotype from a seven-way 15-generation 500-reference inter-continental simulated dataset.

Besides the experiments on three-way admixed individuals, a case study on seven-way admixed individuals was investigated. The goal is to find out how LAI methods perform on individuals admixed from a large number of founder populations, as in a real case scenario, human individuals are involved in multiple population admixture events. Seven-way inter-continental datasets with various reference panel sizes and generations were simulated, and for such challenging datasets, the $r^{2}$ values and the accuracy rates of LAI methods dropped, but Recomb-Mix kept performing well. Supplementary Fig. S4 shows the $r^{2}$ values of the inference results on six LAI methods (values are in Supplementary Table S9). The average accuracy rates are shown in Supplementary Figs S5 and S6 (values are in Supplementary Tables S10 and S11). Figure 3B illustrates an inferred haplotype sample of an admixed individual from the methods. Compared to Fig. 3A, more population labels were mistakenly assigned, as inferring seven-way admixed individuals is a harder task than inferring three-way ones. In general, window-based LAI methods performed better than site-based ones, except Recomb-Mix. Using a window as the smallest unit of inference helps tolerate errors within the window since the population label having the highest estimated probability determines the inference result for the entire window. On the other hand, site-based methods may focus more on a single site’s label, which may affect the inference result of one’s surrounding region when making incorrect inferences, especially if the number of potential population labels is large and the number of reference haplotype templates is limited. Though Recomb-Mix uses a site-based approach, it achieved high accuracy. Allowing individual variations within the same population helps inflate the panel so more reference haplotype templates (e.g. relatives) are available for local site inference.

For a seven-way 200-generation 500-individual inter-continental WGS dataset, FLARE, G-Nomix, and Loter had better $r^{2}$ values than Recomb-Mix. They were 0.1005, 0.0292, and 0.0017 higher than that of Recomb-Mix, respectively. See Supplementary Fig. S7 (values are in Supplementary Table S12). FLARE, G-Nomix, and Loter were claimed as effective LAI methods for identifying distant admixture events, as demonstrated high-resolution accuracy when the admixture event occurs over 100 generations (Dias-Alves *et al.* 2018, Hilmarsson *et al.* 2021, Browning *et al.* 2023). FLARE incorporates the number of generations as a parameter in their model and its value is updated using an iterative expectation maximization (EM) approach to calculate the probabilities of a change of ancestry state for each marker and haplotype. The longer the admixture event occurs, the higher the probability that ancestral segments or tracts having a large length difference appear. This information helps FLARE to update their generation parameter better. On top of G-Nomix’s base module’s classifier, a smoother module is added to refine the inference result. The smoother is a data-driven approach, which learns to capture the distribution of recombination breakpoints. Usually, the distant admixture event has richer information on the distribution of recombinations, which helps G-Nomix’s smoother module improve the accuracy. Loter adopts the bagging technique to generate the averaged result, which avoids putting a strong prior on a particular length of ancestry segment. This helps improve the inference accuracy since the ancestry segments appearing in distant admixture events vary in length.

### 3.3 Intra-continental admixture

Compared to the inter-continental admixture, the LAI on the intra-continental is relatively less studied. The same benchmarks were set up and evaluated as the inter-continental ones. Similar to the results of the inter-continental datasets, Recomb-Mix performed well on the intra-continental datasets. The $r^{2}$ values of the local ancestry inference of six LAI methods on a three-way 15-generation intra-continental simulated dataset are shown in Supplementary Fig. S8 (values are in Supplementary Table S13). The average accuracy rates are shown in Supplementary Fig. S9 (values are in Supplementary Table S14). Overall, the $r^{2}$ values of each method were worse than the ones in the inter-continental datasets. This is expected as the admixture occurring at the intra-continental level generates individuals who resemble each other. Thus, performing LAI on such datasets is more challenging than at the inter-continental level. Recomb-Mix had the best $r^{2}$ value in reference panel sizes 250, 500, and 1000 with 15 generations. For a 250-individual reference panel, the $r^{2}$ value of Recomb-Mix was 0.9299, and the second-best method, G-Nomix, only achieved 0.8560. For a 1000-individual reference panel, the $r^{2}$ values of Recomb-Mix and G-Nomix were close (0.9800 and 0.9820).

The impact of the number of generations on LAI at the intra-continental level was also investigated. Four three-way 500-reference intra-continental simulated datasets with generations 15, 50, 100, and 200 were tested, and the results show both the $r^{2}$ values and accuracy rates tend to decrease as the number of generations grows. See Supplementary Figs S10 (values are in Supplementary Table S15) and S11 (values are in Supplementary Table S16). Recomb-Mix achieved the highest $r^{2}$ value in a 15-generation dataset, while G-Nomix and FLARE achieved better $r^{2}$ values in a 200-generation dataset. Additionally, Loter showed improved performance as the number of generations grew. This result is consistent and observed in other simulated datasets, as G-Nomix, FLARE, and Loter do well in ancestry inference on the cases of distant admixture events.

### 3.4 Efficiency in memory, space, and run time

We examined the run time and maximum amount of memory LAI methods used for their performance on admixed individual haplotypes. The average CPU run time and maximum amount of physical memory that all six LAI methods consumed across different experimental runs are shown in Supplementary Figs S12, S13, and Supplementary Table S17. In general, for the same method, inference on three-way admixed individuals was faster than those on seven-way. This is expected as a seven-way admixture has many more local ancestral segments across the chromosome than ones in a three-way, which costs more time for the inference. All methods showed reasonable run time for an LAI query of an admixed individual haplotype except Loter, which was about 10 or 100 times slower than other methods. SALAI-Net was the fastest method and Recomb-Mix was the runner-up but only took 0.31 and 2.04 more seconds than SALAI-Net in three-way and seven-way datasets. From the memory-consuming perspective, all methods’ memory usage was acceptable, and Recomb-Mix required the smallest amount of memory, 2.44 and 4.13 GB in three-way and seven-way datasets, respectively.

Recomb-Mix has a feature that converts the compact population graph into a Variant Call Format (VCF) file (Danecek *et al.* 2011). Later, they can be reused by Recomb-Mix to save processing time. A compact VCF file is much smaller than the original one since it only contains individual haplotype templates with population-level information. For example, the disk space needed to store a 3-way inter-continental 500-reference panel was decreased from 665 to 13.3 MB (and 1.4 MB for a compressed VCF file). Similarly, for a 7-way panel, the disk space was decreased from 787 to 21.8 MB (and 1.9 MB for a compressed VCF file).

### 3.5 The 1000 Genomes Project and the Human Genome Diversity Project ancestry analysis

To show the scalability and robustness of Recomb-Mix, we estimated the ancestry proportions from the inferred local ancestries for the populations in the 1000 Genomes Project (TGP) data (Byrska-Bishop *et al.* 2022) using the four founder populations (Africans, Admixed Americans, East Asians, and Europeans) as the reference panel from the Human Genome Diversity Project (HGDP) data (Bergström *et al.* 2020). Similarly, we estimated the ancestry proportions for the populations in the HGDP data using the four founder populations (Africans, East Asians, Europeans, and Native Americans) from the TGP data. We merged two Chromosome 18 datasets (TGP with 3 457 645 markers and HGDP with 2 127 412 markers), yielding 1 165 399 intersected markers. Then the merged dataset was phased using Beagle 5.4 and the individuals were assigned the population labels provided by their original datasets.

**Figure 4.**
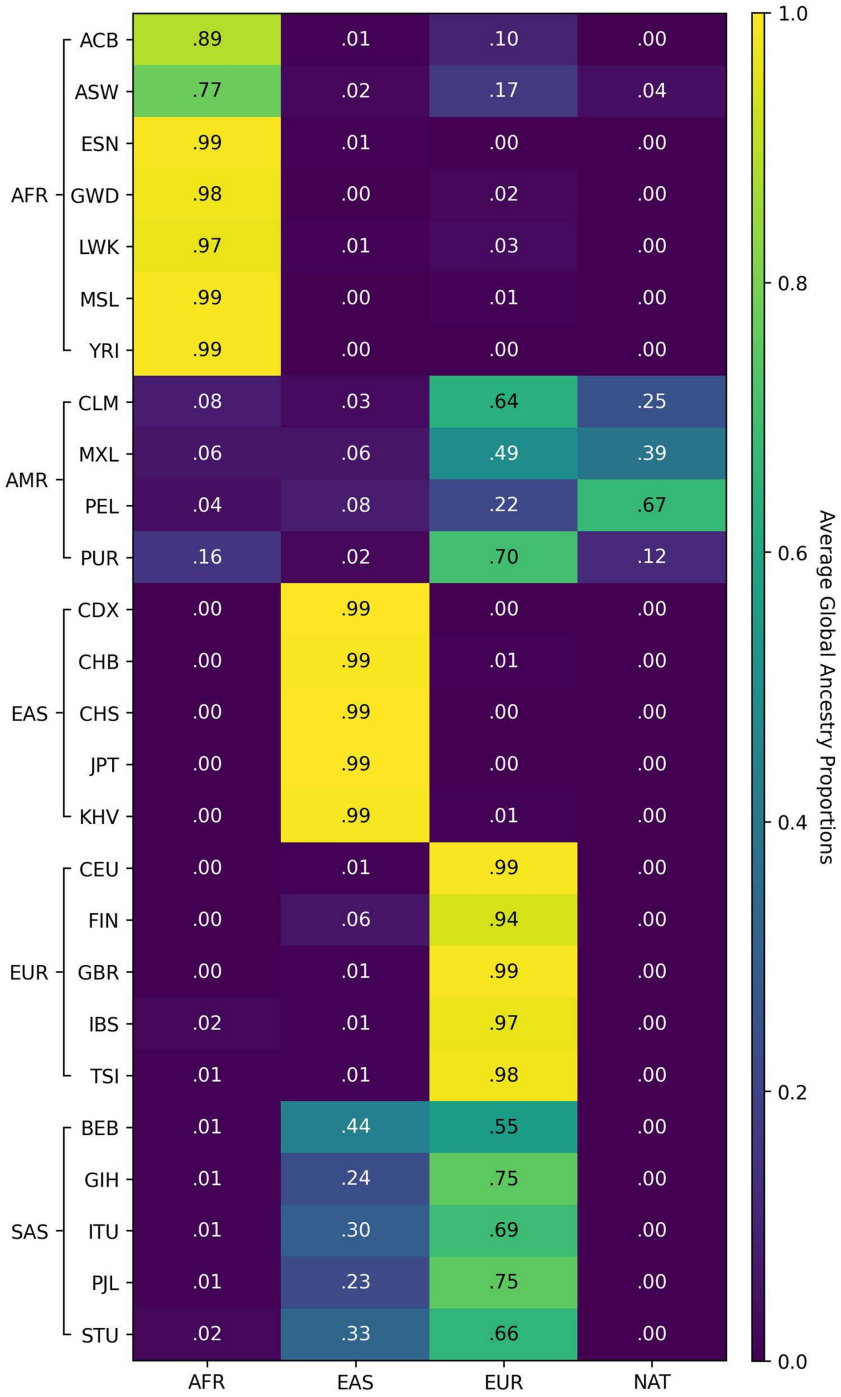
The average global ancestry proportions in the 1000 Genomes Project Chromosome 18 data using four reference ancestries from the Human Genome Diversity Project data. Descriptions of the populations are in Supplementary Table S2.

Like FLARE (Browning *et al.* 2023), we calculated the global ancestry composition by averaging the estimated local ancestry proportions across the genome. Figure 4 is Recomb-Mix’s ancestry inference result on the TGP dataset that is generally consistent with exceptions. Similar results using other LAI methods are available in Supplementary Fig. S14. For African individuals who reside in the African continent, at least 97% segment was labeled as African on average. For ACB and ASW individuals (located in the Caribbean and America), a small portion of the segment was labeled as non-African due to their admixed backgrounds. Most segments of American individuals were labeled as mixed percentages of Europeans and Native Americans. South Asian individuals were labeled as mixed percentages of East Asian and European. East Asian and European individuals had at least 99% and 94% segments labeled as East Asian and European, respectively.

**Figure 5.**
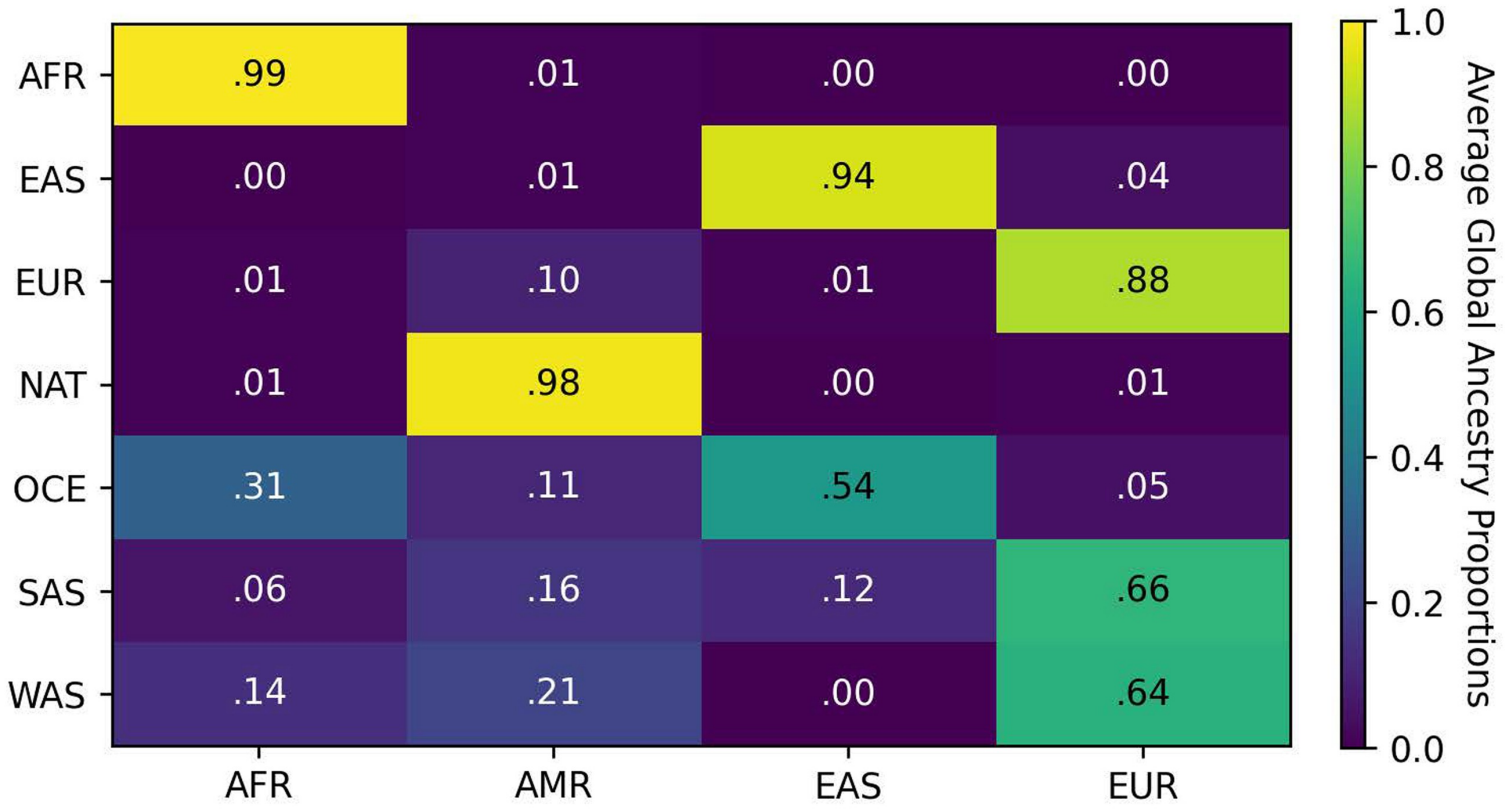
The average global ancestry proportions in the Human Genome Diversity Project Chromosome 18 data using four reference ancestries from the 1000 Genomes Project data. Descriptions of the populations are in Supplementary Table S2.

The ancestry inference results for the HGDP dataset generally align with the expected ancestry proportions for each population in demographic history. Figure 5 shows that Recomb-Mix predicted African, East Asian, European, and native American individuals have 99%, 94%, 88%, and 98% segments matched expected ancestries. For the Oceanian individuals, the segments were decomposed into mixed ancestries, primarily East Asian and African. For South Asian and West Asian individuals, the segments were inferred as mixed and mainly consisted of European ancestry. Interpreting ancestry inference results was more challenging when certain populations shared the same ancient ancestry which was not presented in the reference panel. For example, recent genetic evidence suggests that EUR, WAS, and SAS may share some Yamnaya DNA (Narasimhan *et al.* 2019, Lazaridis *et al.* 2022), which might be part of the causes of our results of possible shared (about 10%) ancient ancestry among AMR, EUR, OCE, SAS, and WAS. Similar behaviors were observed on other LAI methods, such as G-Nomix and SALAI-Net (see Supplementary Fig. S15). Recomb-Mix was forced to give a single LAI call for these regions since the inference results were based on the given reference panels. If the ancient population were not in the reference panel, the segment would be labeled as the population closest to the ancient one.

### 3.6 Discrete ancestry informative markers in Recomb-Mix

Since dAIMs are operationally defined and depend on some random chance of whether an allele is present in the reference panel for the population or not, we investigated the availability of dAIMs in real-world datasets and the usability of dAIMs in Recomb-Mix’s LAI process (Supplementary Results S4.1). We demonstrated that dAIMs share ancestry-specific signals and are densely available on real-world datasets. We also showed that the accuracy of Recomb-Mix’s inference differs among populations, but there is no strong correlation with their signal strengths. Therefore, even though collapsing nodes reduced the information in the original panel, the remaining information in dAIMs is sufficient for Recomb-Mix to make good-quality ancestry calls.

### 3.7 Robustness and ablation study

We conducted several additional experiments to assess the robustness of Recomb-Mix and other methods in their performance for LAI. These experiments included admixture with uneven proportions of founders and references, admixture with ancestry misspecification panel, and admixture with phasing error. The results indicated that all LAI methods were impacted by these imperfect situations but they still performed well (Supplementary Results S4.2–S4.4).

We investigated the components that contribute to Recomb-Mix’s ancestry inference process. It is found that setting the within-population template change penalty to zero significantly improves Recomb-Mix’s performance since this setting effectively helps differentiate populations during the inference process. We also found that filtering out certain allele values of sites based on their minor allele frequencies can alleviate the situation when the reference panel has no or very limited dAIMs presented (Supplementary Results S4.5).

## 4 Discussion

We presented a new local ancestry inference method named Recomb-Mix, based on a simplified Li and Stephens model formulating LAI as a graph optimization problem. By not considering recombination penalties within populations, Recomb-Mix shows promising LAI results under various conditions. A compact population graph also helps Recomb-Mix process LAI effectively and efficiently, as it retains marker informativeness for ancestry through dAIMs. Furthermore, it is convenient to store the reference panel as a compact population graph on disk, which takes up little space for future ancestry inference without a re-transformation process. Recomb-Mix is competitive with other state-of-the-art LAI methods in accuracy and computational performance and is applicable to real genomic datasets.

As a site-based LAI method, Recomb-Mix is designed to exploit the site-level information to achieve superior accuracy, especially at the intra-continental level. The number of dAIMs in intra-continental admixed individuals is less than those in inter-continental admixed individuals, as intra-dAIMs are a subset of inter-dAIMs. Window-based LAI methods may find it difficult to achieve high accuracy during the intra-level ancestry inference. There is a higher probability for each window containing multiple intra-dAIMs than that for inter-dAIMs. Since the window is the smallest unit representing one ancestral source, windows having intra-dAIMs representing different populations may easily misrepresent the inference result. Decreasing the window size may mitigate the situation, with the potential computational burden. However, its lower bound is one site per window, i.e. site-based.

Despite the high accuracy rates demonstrated, Recomb-Mix has limitations of not considering the disparate genetic maps across populations, the allele frequencies, or genotyping and phasing errors. Thus, other complementary methods may be useful for a well-specified model. FLARE takes optional parameters such as minor allele frequency and number of generations since admixture. It may perform well if these biological parameters are correctly estimated for the model. G-Nomix offers a few pre-trained models, which may be helpful if one were pre-trained specifically for the given model. SALAI-Net may be a good choice as it uses a pre-trained model that is generalized and applicable to any species and any set of ancestries.

Although Recomb-Mix was not designed to handle erroneous panels, both genotyping errors and phasing errors seem to have no large impact on the inference results. However, other types of noisy data, such as individuals incorrectly classified into the wrong population in the reference panel, may influence the LAI result. If the error rate is high, a pre-processing step may be needed to correct the noisy data panel before making the inference. Currently, our approach only reports the single best path in the collapsed population graph, i.e. the one with parsimony scores. This limitation may compromise robustness and accuracy. A standard practice has been returning a number of high-probability solutions or sampling the solutions according to the posterior of a Bayesian framework. In future studies, we plan to consider this practice and explore alternative ways of collapsing the graph to minimize noise and retain more relevant ancestry information from the reference panel. These optimizations might involve selecting markers that are present in multiple populations but with different allele frequencies, which could further enhance Recomb-Mix’s performance and enable the provision of uncertainty estimates for ancestry inference results.

## Supplementary Material

btaf227_Supplementary_Data

## Data Availability

The Recomb-Mix code and simulations are available at https://github.com/ucfcbb/Recomb-Mix.
