# Supplementary material for: Recomb-Mix: fast and accurate local ancestry inference
Source: Bioinformatics. 2025 Jul 15;41(Suppl 1):i180–8. doi: 10.1093/bioinformatics/btaf227 (PMC12261469; doi:10.1093/bioinformatics/btaf227)
Supplement: btaf227_Supplementary_Data [file btaf227_supplementary_data.pdf]

Supplementary Materials for "Recomb-Mix: fast and accurate local ancestry inference"

Yuan Wei[1], Degui Zhi[2], and Shaojie Zhang[1]

[1]Department of Computer Science
University of Central Florida, Orlando, FL, USA

[2]McWilliams School of Biomedical Informatics
University of Texas Health Science Center at Houston, Houston, TX, USA

S1 Supplementary Algorithm

Recomb-Mix adopts a dynamic programming approach to find the optimal solution for LAI. For a query haplotype q, P_j^i is defined as a threading path with the minimum penalty score from the start node s to node s_j^i. C_j^i, the penalty score of P_j^i, can be computed as:

$$C_j^i = d(a(q_j), a(s_j^i)) + \min \begin{cases} C_{j-1}^{x_{j-1}^{l(s_j^i)}} \\ C_{j-1}^{y_{j-1}} + wr(l(s_{j-1}^{y_{j-1}}), l(s_j^i)), \end{cases} \tag{1}$$

where $x_{j-1}^{l(s_j^i)}$ is the node index with the minimum penalty score of ancestry $l(s_j^i)$ at site $j-1$, and y_{j-1} is the node index with the minimum penalty score over all the ancestries at site $j-1$.

The solution to the original problem is equivalent to computing a threading path from s to e with the minimum penalty score. The number of sites in G' is n, and the number of nodes for each site is $|S'_j|$, where $|S'_j| \in [p, 2p]$. Thus, there are at most $n \times 2p$ nodes in G', and the number of subproblems is $O(np)$. Each subproblem is evaluated once by using the objective function (1), and the evaluation takes a constant amount of time as the computed penalty scores are accessible in C for each node. For each site j, C stores the minimum penalty score ending at node s_j^i in C_j^i. x_j^k stores the node index with the minimum penalty score having ancestry label k. y_j stores the node index with the minimum penalty score over all the ancestries. T stores information for traceback. Particularly, T_j^i stores the index of a node in site $j-1$ such that $s_{j-1}^{T_j^i}$ occurs directly before s_j^i in P_j^i.

Algorithm S1 is the pseudocode of Recomb-Mix's dynamic programming approach. For each node s_j^i, two nodes are evaluated to compute the minimum penalty score: the node with the same ancestral label having the minimum score and the node having the overall minimum score at site $j-1$. For nodes with identical penalty scores, the first observed is selected as part of the optimal solution. It is not necessary to evaluate nodes other than these two since nodes with different ancestral labels receive a constant template change penalty. i.e., the template change penalty $r(l(s_{j-1}^{y_{j-1}}), l(s_j^i))$ is 0 if $l(s_{j-1}^{y_{j-1}}) = l(s_j^i)$ and constant otherwise. Thus, the time complexity of computing penalty scores on G' is $O(np)$. From the space perspective, C costs $2p \times n$ space, x costs $p \times n$ space, and y costs n space. Overall, the space complexity is $O(np)$.

Algorithm S1 Compute Penalty Scores and Traceback Ancestral Labels

// Compute penalty scores
// Compute scores for site 1
for i from 1 to $|S'_j|$ **do**
    $C_j^i = d(a(q_j), a(s_j^i))$
// Compute scores for the remaining sites
for j from 2 to n **do**
    for i from 1 to $|S'_j|$ **do**
        $k = l(s_j^i)$
        // Compute minimum score and store the corresponding node index at site $j - 1$
        $C_j^i = d(a(q_j), a(s_j^i)) + \min(C_{j-1}^{x_{j-1}^k}, C_{j-1}^{y_{j-1}} + wr(l(s_{j-1}^{y_{j-1}}), k))$
        if $C_j^i = C_{j-1}^{x_{j-1}^k} + d(a(q_j), a(s_j^i))$ **then**
            $T_j^i = x_{j-1}^k$
        else
            $T_j^i = y_{j-1}$
        // update minimum scores for each ancestry at site j
        if $C_j^{x_j^k} > C_j^i$ **then**
            $x_j^k = i$
        // update overall minimum scores at site j
        if $C_j^{y_j} > C_j^i$ **then**
            $y_j = i$
// Traceback ancestral labels
$k = 1$
for i from 2 to $|S'_n|$ **do**
    if $C_n^i < C_n^k$ **then**
        $k = i$
for j from n to 1 **do**
    Report $l(s_j^k)$ as q_j's ancestral label
    $k = T_j^k$

S2 Supplementary Tables

Features	HapMix	FLARE	Loter	Recomb-Mix
Within population transition probability / template change penalty	Recombination parameter	the probability of the position derived from an ancestry, the probability of the position derived from a haplotype, and ancestry-specific switch rate	1	0
Across population transition probability / template change penalty	Recombination parameter and miscopying parameter	the probability of the position derived from an ancestry and the probability of the position derived from a haplotype	1	Recombination penalty
Emission probability / mismatch penalty	Mutation parameter	Mismatch rate	Mismatch penalty	Mismatch penalty
Parameterization of transition probability	$1 - e^x$, where x is the product of genetic distance and recombination parameter	$1 - e^{x'}$, where x' is the product of genetic distance and number of generations	Bootstrap aggregation (bagging)	Weight
Panel Compression	$O(nm)$, no compression	$O(nm')$, composite reference haplotypes, constructed from stitching Identity-By-State (IBS) segments from the panel (m' depends on the number and locations of IBS segments)	$O(nm)$, no compression	$O(np)$, compact population graph, constructed by collapsing the nodes having the same allele value and ancestral label per site

Table S1: Feature summary of HapMix, FLARE, Loter, and Recomb-Mix. All methods incorporate the emission probability and the transition probability inspired by the Li and Stephens model. HapMix, FLARE, and Recomb-Mix capture the difference between inter-population and intra-population transition probabilities, and utilize genetic information in their parameterizations and scoring functions. Recomb-Mix and FLARE extract ancestry information from compressed reference panels. n is the number of sites, m is the number of individual haplotypes, and p is the number of distinct ancestral populations in the panel.

Population Code	Population Description
ACB	African Caribbeans in Barbados
AFR	African
AMR	Admixed American
ASW	Americans of African Ancestry in SW USA
BEB	Bengali from Bangladesh
CDX	Chinese Dai in Xishuangbanna, China
CEU	Utah Residents (CEPH) with Northern and Western European Ancestry
CHB	Han Chinese in Beijing, China
CHS	Southern Han Chinese
CLM	Colombians from Medellin, Colombia
EAS	Eastern Asian
ESN	Esan in Nigeria
EUR	European
FIN	Finnish in Finland
GBR	British in England and Scotland
GIH	Gujarati Indian from Houston, Texas
GWD	Gambian in Western Divisions in the Gambia
IBS	Iberian Population in Spain
ITU	Indian Telugu from the UK
JPT	Japanese in Tokyo, Japan
KHV	Kinh in Ho Chi Minh City, Vietnam
LWK	Luhya in Webuye, Kenya
MSL	Mende in Sierra Leone
MXL	Mexican Ancestry from Los Angeles USA
NAT	Native American
OCE	Oceanian
PEL	Peruvians from Lima, Peru
PJL	Punjabi from Lahore, Pakistan
PUR	Puerto Ricans from Puerto Rico
SAS	Central/South Asian
STU	Sri Lankan Tamil from the UK
TSI	Toscani in Italia
WAS	Middle Eastern/Western Asian
YRI	Yoruba in Ibadan, Nigeria

Table S2: Population code and description in the 1000 Genomes Project (TGP) and the Human Genome Diversity Project (HGDP) data.

Method	Parameters
FLARE	min-mac=0 min-maf=0 ref=reference.vcf gt=query.vcf map=genetic_map.txt ref-panel=reference_population_label.txt out=output_basename
G-Nomix	query.vcf output_folder 18 False genetic_map.txt reference.vcf reference_population_label.txt default_config.yaml
Loter	-r reference_population_1.vcf reference_population_2.vcf reference_population_3.vcf -a query.vcf -f vcf -o output_result.txt -n 1 -v
MOSAIC	ADMIX Data/ -a 3 -n 200 -c 18 -p "AFR EAS EUR" -m 1 –nophase FALSE –singlePI TRUE
Recomb-Mix	-p reference.vcf -q query.vcf -a reference_population_label.txt -g genetic_map.txt -o output_folder
RFMix	-f query.vcf -r reference.vcf -m reference_population_label.txt -g genetic_map.txt -o output_basename –chromosome=18
SALAI-Net	–model-cp models/main_model/models/best_model.pth -q query.vcf -r reference.vcf -m reference_population_label.txt -o output_folder

Table S3: Parameters of LAI methods used for performance analysis of the experiments. For Loter, it does not take sample map file "reference_population_label.txt" and reference file "reference.vcf". The reference file needs to be split into multiple reference file(s) per population as the input (e.g., reference_population_1.vcf, reference_population_2.vcf, etc.). For MOSAIC, the input files need to be converted to snpfile.CHR, rates.CHR, sample.names, and POPgenofile.CHR for each admixed and reference VCF files. All the files are located in the "Data" folder.

Method	100	250	500	1,000
FLARE	0.8664	0.9559	0.9894	0.9944
G-Nomix	0.9681	0.9882	0.9979	0.9989
Loter	0.8389	0.9482	0.9817	0.9909
Recomb-Mix	0.9919	0.9972	0.9995	0.9989
RFMix	0.8046	0.9733	0.9964	0.9982
SALAI-Net	0.9480	0.9936	0.9970	0.9970

Table S4: The squared Pearson's correlation coefficient r^2 with the reference panel sizes 100, 250, 500, and 1,000 of the three-way 15-generation inter-continental simulated datasets on FLARE, G-Nomix, Loter, Recomb-Mix, RFMix, and SALAI-Net. Markers were filtered with minor allele frequency ≤ 0.005 and minor allele count ≤ 50.

Method	15	50	100	200
FLARE	0.9894	0.9551	0.9159	0.8499
G-Nomix	0.9979	0.9813	0.9492	0.9204
Loter	0.9817	0.9461	0.8790	0.8130
Recomb-Mix	0.9995	0.9912	0.9725	0.9296
RFMix	0.9964	0.9451	0.7807	0.7520
SALAI-Net	0.9970	0.9762	0.8495	0.6435

Table S5: The squared Pearson's correlation coefficient r^2 with the generations 15, 50, 100, and 200 of the three-way 500-reference inter-continental simulated datasets on FLARE, G-Nomix, Loter, Recomb-Mix, RFMix, and SALAI-Net. Markers were filtered with minor allele frequency ≤ 0.005 and minor allele count ≤ 50.

Method	100	250	500	1,000
FLARE	62.65	76.09	87.42	98.14
G-Nomix	86.63	92.26	96.58	98.24
Loter	62.82	75.91	84.69	91.99
Recomb-Mix	97.96	98.44	98.93	99.10
RFMix	72.22	92.85	96.36	98.14
SALAI-Net	86.69	95.47	96.28	96.72

Table S6: The average accuracy rates with the reference panel sizes 100, 250, 500, and 1,000 of the three-way 15-generation inter-continental simulated datasets on FLARE, G-Nomix, Loter, Recomb-Mix, RFMix, and SALAI-Net.

Method	15	50	100	200
FLARE	87.42	71.32	62.59	52.12
G-Nomix	96.58	86.71	80.63	73.04
Loter	84.69	71.08	63.92	54.60
Recomb-Mix	98.93	95.15	91.47	80.76
RFMix	96.36	81.79	67.05	56.26
SALAI-Net	96.28	89.19	74.84	51.72

Table S7: The average accuracy rates with the generations 15, 50, 100, and 200 of the three-way 500-reference inter-continental simulated datasets on FLARE, G-Nomix, Loter, Recomb-Mix, RFMix, and SALAI-Net.

**Method**	**20**	**50**	**100**	**250**	**500**	**1,000**
FLARE	50.21	59.59	62.65	76.09	87.42	93.32
G-Nomix	60.92	76.50	86.63	92.26	96.58	98.24
Loter	52.75	62.02	62.82	75.91	84.69	91.99
MOSAIC	48.84	70.06	79.73	82.01	87.51	84.12
Recomb-Mix	62.85	94.45	97.96	98.35	98.95	98.98
RFMix	51.28	61.08	72.22	92.85	96.36	98.14
SALAI-Net	52.72	71.39	86.69	95.47	96.28	96.72

Table S8: The average accuracy rates with the reference panel sizes 20, 50, 100, 250, 500, and 1,000 of the three-way 15-generation inter-continental simulated datasets on FLARE, G-Nomix, Loter, MOSAIC, Recomb-Mix, RFMix, and SALAI-Net.

**Method**	**250**	**500**	**1,000**
FLARE	0.6484	0.8509	0.8788
G-Nomix	0.7563	0.9100	0.9812
Loter	0.6457	0.7705	0.8240
Recomb-Mix	0.9544	0.9798	0.9904
RFMix	0.5107	0.8699	0.9002
SALAI-Net	0.7407	0.9535	0.9740

Table S9: The squared Pearson's correlation coefficient r^2 with the reference panel sizes 250, 500, and 1,000 of the seven-way 15-generation inter-continental simulated datasets on FLARE, G-Nomix, Loter, Recomb-Mix, RFMix, and SALAI-Net. Markers were filtered with minor allele frequency ≤ 0.005 and minor allele count ≤ 50. The reference panel size 100 case was not included because the number of markers was too small and may have influenced the outcome after the filtering.

**Method**	**100**	**250**	**500**	**1,000**
FLARE	31.89	41.62	53.41	62.93
G-Nomix	38.50	54.48	80.88	88.14
Loter	32.16	39.88	48.05	58.68
Recomb-Mix	86.02	90.33	94.40	94.34
RFMix	39.93	56.06	77.56	80.35
SALAI-Net	38.99	60.44	85.85	84.42

Table S10: The average accuracy rates with the reference panel sizes 100, 250, 500, and 1,000 of the seven-way 15-generation inter-continental simulated datasets on FLARE, G-Nomix, Loter, Recomb-Mix, RFMix, and SALAI-Net.

Method	15	50	100	200
FLARE	53.41	37.23	30.85	27.00
G-Nomix	80.88	59.58	54.13	43.62
Loter	48.05	36.59	32.58	32.29
Recomb-Mix	94.40	83.27	71.01	42.27
RFMix	77.56	47.41	40.07	34.20
SALAI-Net	85.85	48.34	35.81	35.05

Table S11: The average accuracy rates with the generations 15, 50, 100, and 200 of the seven-way 500-reference inter-continental simulated datasets on FLARE, G-Nomix, Loter, Recomb-Mix, RFMix, and SALAI-Net.

Method	15	50	100	200
FLARE	0.8509	0.8323	0.7831	0.6558
G-Nomix	0.9100	0.8322	0.8462	0.5845
Loter	0.7705	0.7694	0.6970	0.5570
Recomb-Mix	0.9798	0.9268	0.8728	0.5553
RFMix	0.8699	0.6028	0.5968	0.4187
SALAI-Net	0.9535	0.7469	0.5457	0.4869

Table S12: The squared Pearson's correlation coefficient r^2 with the generations 15, 50, 100, and 200 of the seven-way 500-reference inter-continental simulated datasets on FLARE, G-Nomix, Loter, Recomb-Mix, RFMix, and SALAI-Net. Markers were filtered with minor allele frequency ≤ 0.005 and minor allele count ≤ 50.

Method	250	500	1,000
FLARE	0.7232	0.7538	0.9273
G-Nomix	0.8560	0.9235	0.9820
Loter	0.6441	0.7264	0.8794
Recomb-Mix	0.9299	0.9625	0.9800
RFMix	0.7180	0.8024	0.9077
SALAI-Net	0.7910	0.9081	0.9687

Table S13: The squared Pearson's correlation coefficient r^2 with the reference panel sizes 250, 500, and 1,000 of the three-way 15-generation intra-continental simulated datasets on FLARE, G-Nomix, Loter, Recomb-Mix, RFMix, and SALAI-Net. Markers were filtered with minor allele frequency ≤ 0.005 and minor allele count ≤ 50. The reference panel size 100 case was not included because the number of markers was too small and may have influenced the outcome after the filtering.

Method	100	250	500	1,000
FLARE	40.98	56.21	64.62	79.56
G-Nomix	42.71	70.91	81.83	91.82
Loter	39.42	53.57	59.28	77.15
Recomb-Mix	85.55	89.76	91.33	92.38
RFMix	41.81	69.57	75.66	86.04
SALAI-Net	49.83	77.70	86.45	89.38

Table S14: The average accuracy rates with the reference panel sizes 100, 250, 500, and 1,000 of the three-way 15-generation intra-continental simulated datasets on FLARE, G-Nomix, Loter, Recomb-Mix, RFMix, and SALAI-Net.

Method	15	50	100	200
FLARE	0.7538	0.7768	0.7784	0.7595
G-Nomix	0.9235	0.7912	0.7992	0.8048
Loter	0.7264	0.6406	0.6978	0.6993
Recomb-Mix	0.9625	0.8506	0.7930	0.7428
RFMix	0.8024	0.7072	0.6686	0.6035
SALAI-Net	0.9081	0.7281	0.6269	0.4679

Table S15: The squared Pearson's correlation coefficient r^2 with the generations 15, 50, 100, and 200 of the three-way 500-reference intra-continental simulated datasets on FLARE, G-Nomix, Loter, Recomb-Mix, RFMix, and SALAI-Net. Markers were filtered with minor allele frequency ≤ 0.005 and minor allele count ≤ 50.

Method	15	50	100	200
FLARE	64.62	50.42	48.66	44.15
G-Nomix	81.83	59.04	57.46	54.07
Loter	59.28	46.56	48.12	47.02
Recomb-Mix	91.33	81.54	74.76	72.46
RFMix	75.66	67.16	59.85	46.09
SALAI-Net	86.45	69.18	61.89	54.38

Table S16: The average accuracy rates with the generations 15, 50, 100, and 200 of the three-way 500-reference intra-continental simulated datasets on FLARE, G-Nomix, Loter, Recomb-Mix, RFMix, and SALAI-Net.

**Method**	**3-way Run Time (second)**	**7-way Run Time (second)**	**3-way Memory (GB)**	**7-way Memory (GB)**
FLARE	2.60	24.69	23.75	23.75
G-Nomix	3.46	14.60	46.26	48.22
Loter	163.45	276.99	6.08	12.94
Recomb-Mix	1.41	4.37	2.44	4.13
RFMix	18.36	46.06	13.77	40.24
SALAI-Net	1.10	2.33	17.16	28.55

Table S17: The average run time (second) and maximum amount of physical memory (GB) of LAI methods FLARE, G-Nomix, Loter, Recomb-Mix, RFMix, and SALAI-Net for querying local ancestry information of an admixed individual haplotype. Values were averaged from runs of inter- and intra-continental, with reference panel sizes 100, 250, 500, and 1,000 and generations 15, 50, 100, and 200. All methods were tested on a single-node machine with an Intel Xeon Gold 5215 2.50 GHz processor and 200 gigabytes of RAM.

S3 Supplementary Figures

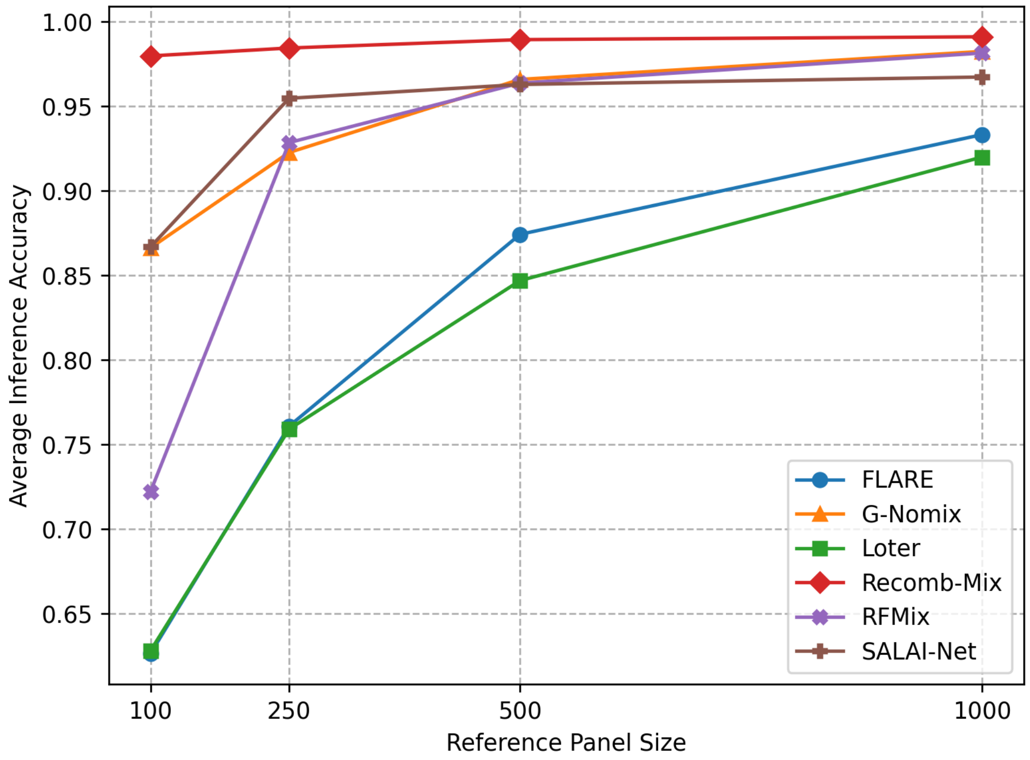

Figure S1: The average accuracy rates with the reference panel sizes 100, 250, 500, and 1,000 of the three-way 15-generation inter-continental simulated datasets on FLARE, G-Nomix, Loter, Recomb-Mix, RFMix, and SALAI-Net.

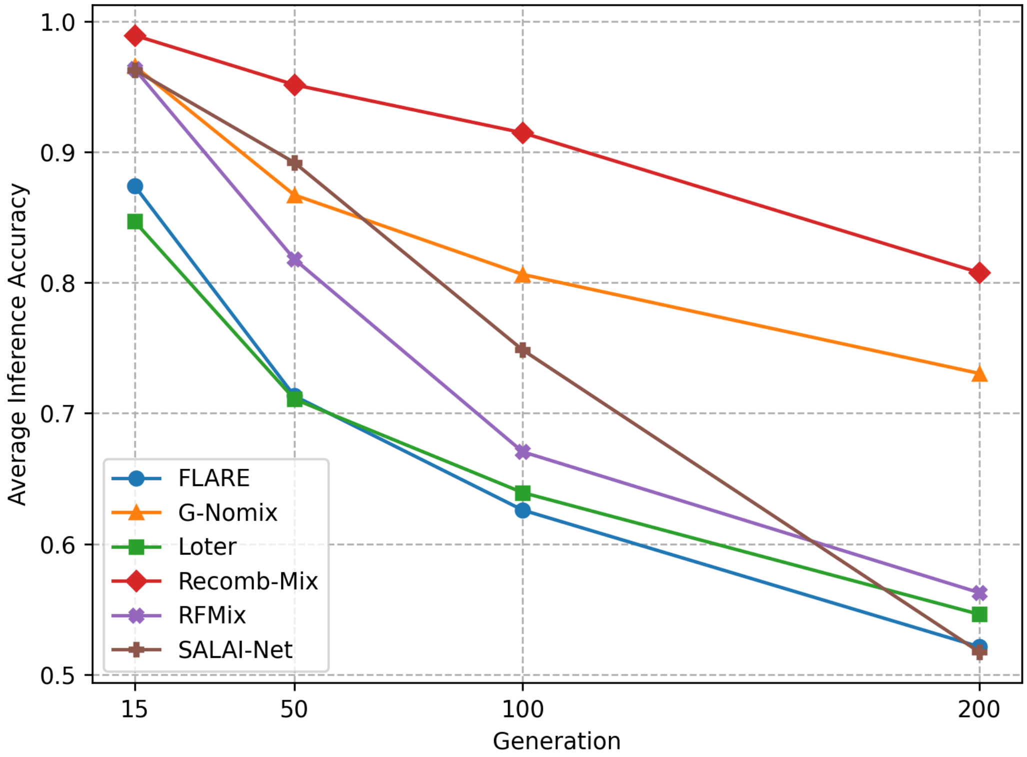

Figure S2: The average accuracy rates with the generations 15, 50, 100, and 200 of the three-way 500-reference inter-continental simulated datasets on FLARE, G-Nomix, Loter, Recomb-Mix, RFMix, and SALAI-Net.

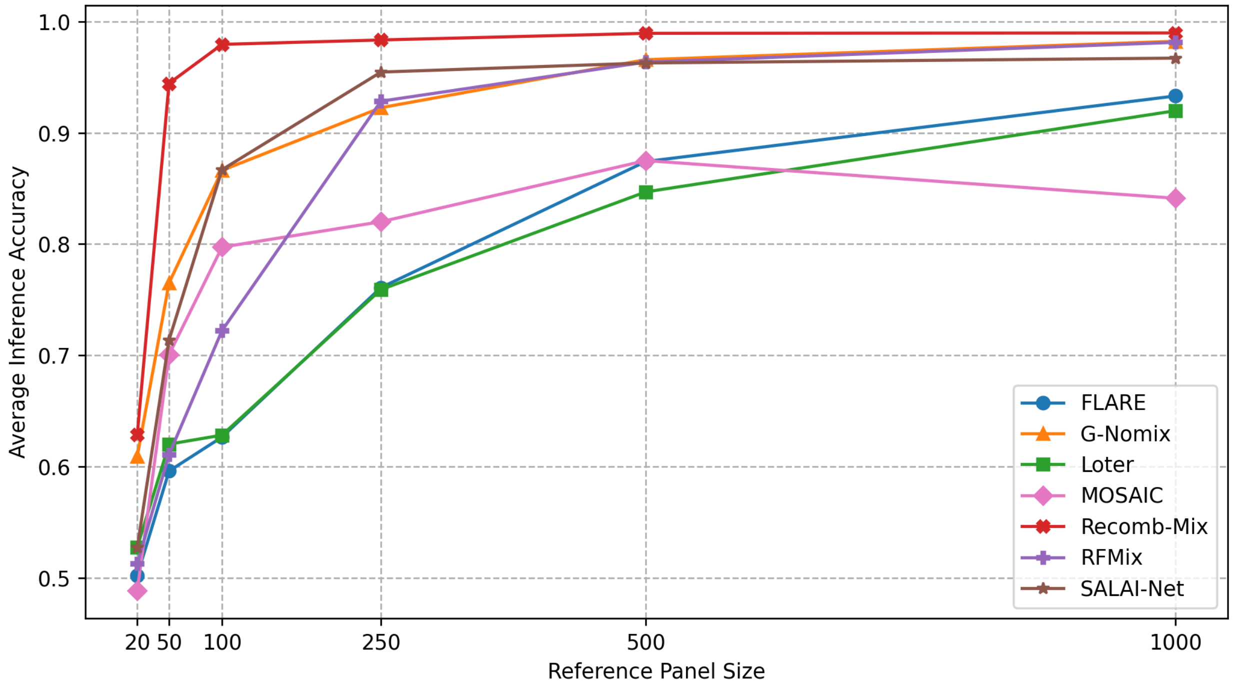

Figure S3: The average accuracy rates with the reference panel sizes 20, 50, 100, 250, 500, and 1,000 of the three-way 15-generation inter-continental simulated datasets on FLARE, G-Nomix, Loter, MOSAIC, Recomb-Mix, RFMix, and SALAI-Net.

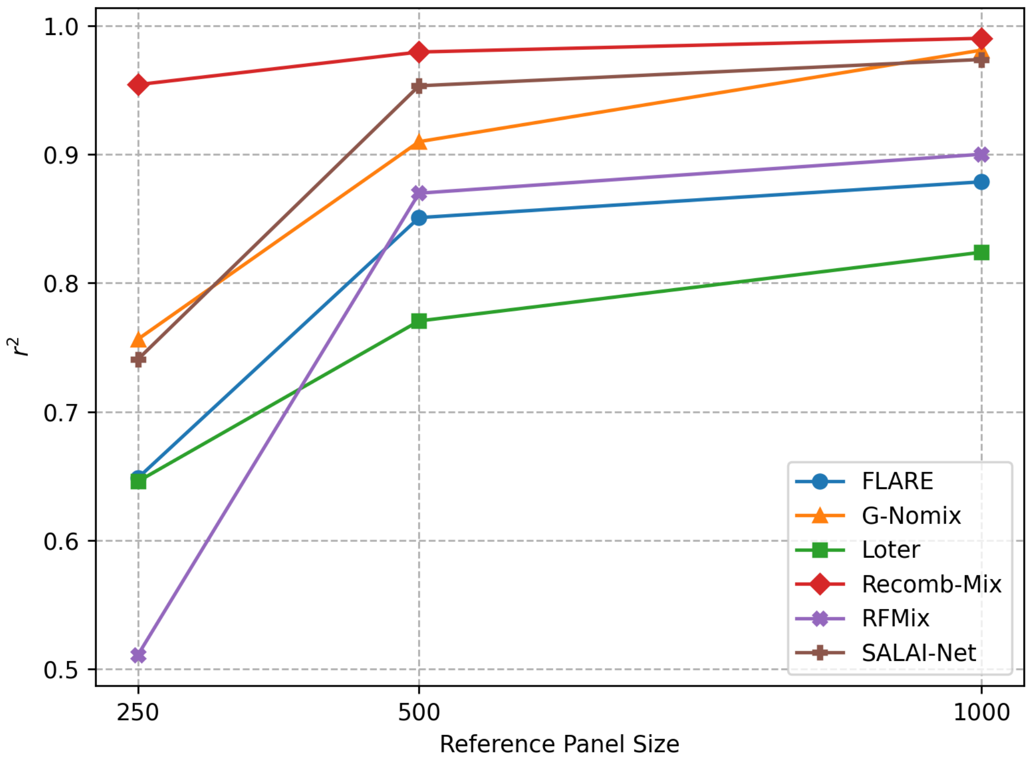

Figure S4: The squared Pearson's correlation coefficient r^2 with the reference panel sizes 250, 500, and 1,000 of the seven-way inter-continental simulated datasets on FLARE, G-Nomix, Loter, Recomb-Mix, RFMix, and SALAI-Net. Markers were filtered with minor allele frequency ≤ 0.005 and minor allele count ≤ 50. The reference panel size 100 case was not included because the number of markers was too small and may have influenced the outcome after the filtering.

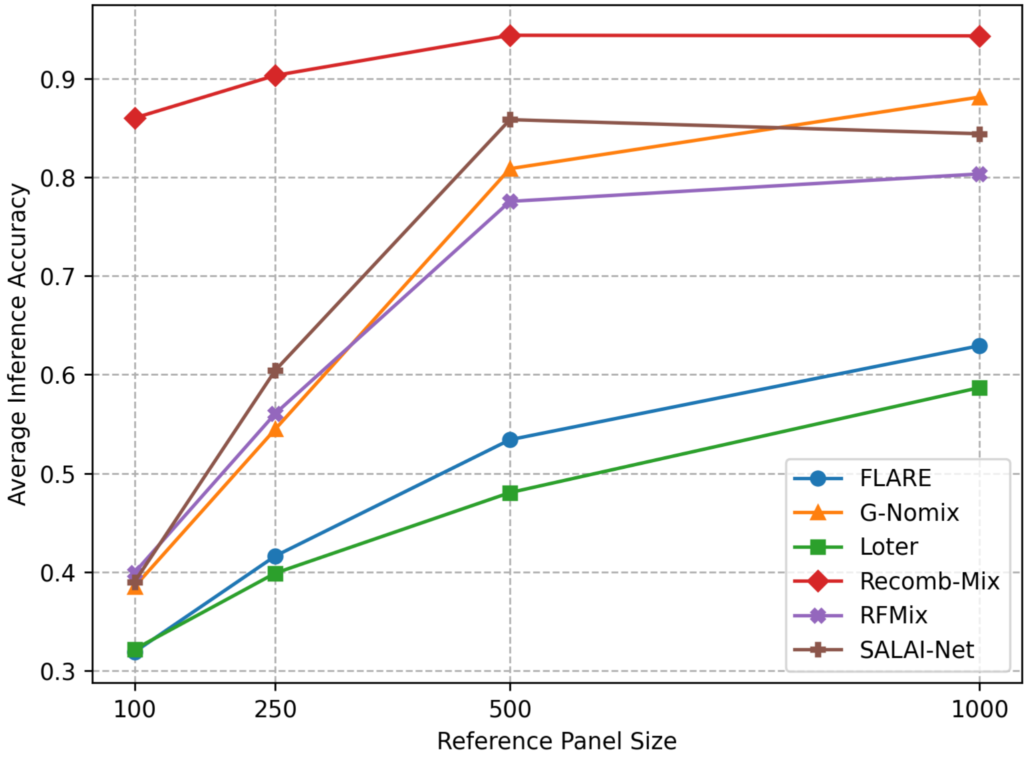

Figure S5: The average accuracy rates with the reference panel sizes 100, 250, 500, and 1,000 of the seven-way 15-generation inter-continental simulated datasets on FLARE, G-Nomix, Loter, Recomb-Mix, RFMix, and SALAI-Net.

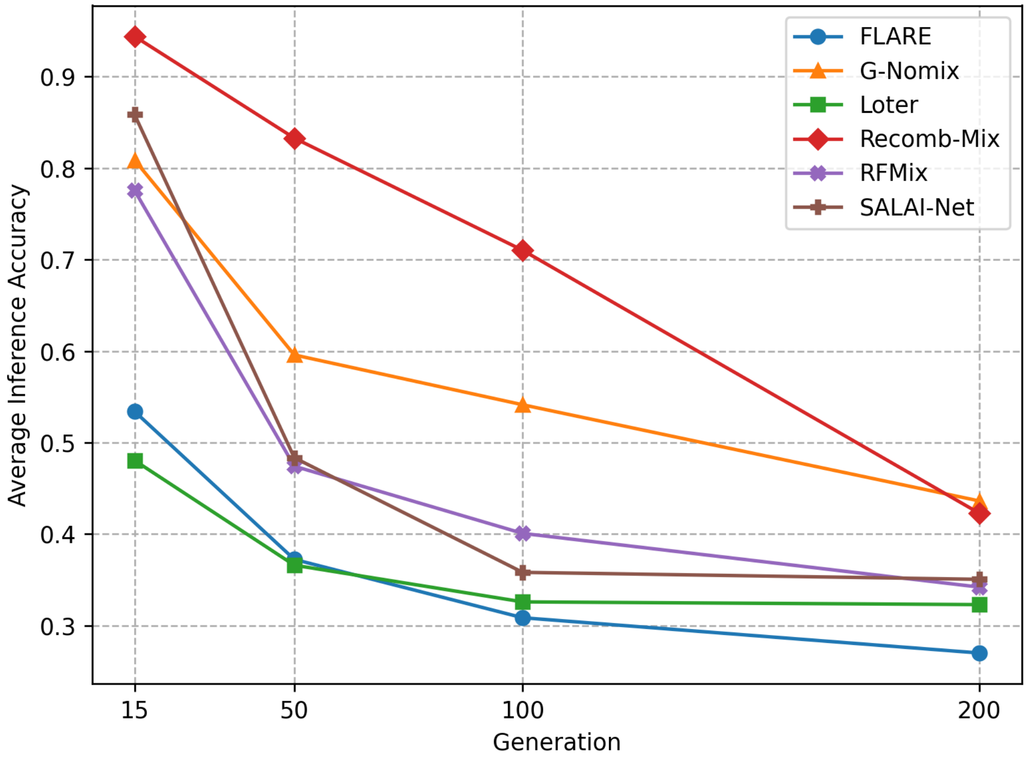

Figure S6: The average accuracy rates with the generations 15, 50, 100, and 200 of the seven-way 500-reference inter-continental simulated datasets on FLARE, G-Nomix, Loter, Recomb-Mix, RFMix, and SALAI-Net.

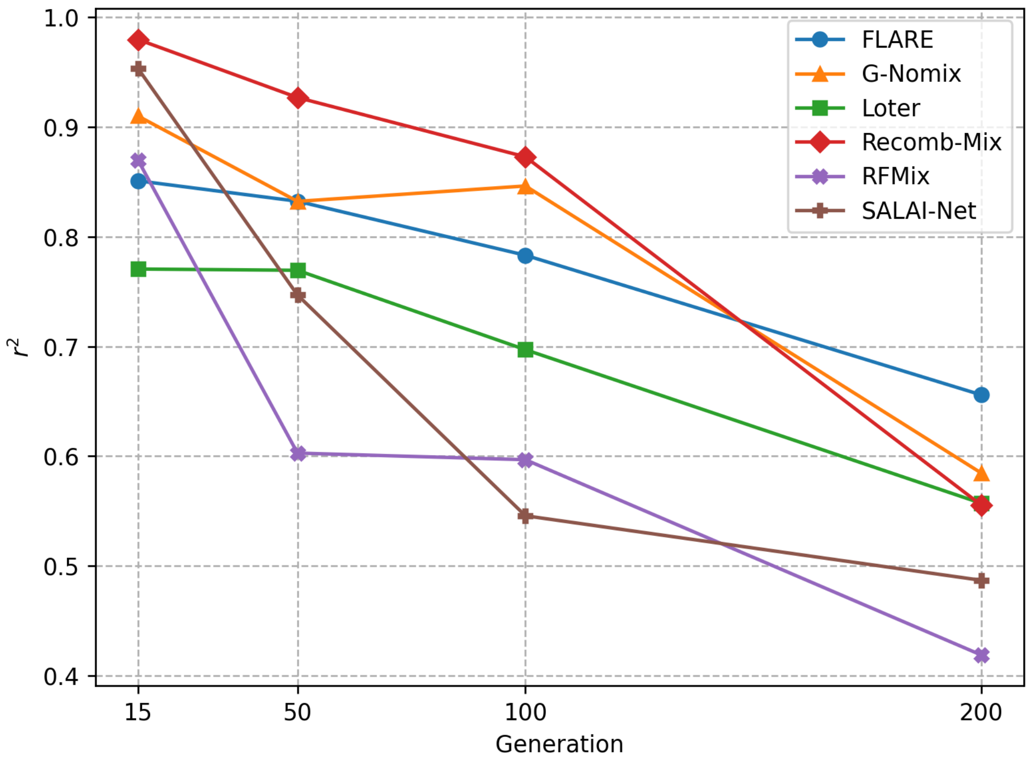

Figure S7: The squared Pearson's correlation coefficient r^2 with the generations 15, 50, 100, and 200 of the seven-way inter-continental simulated datasets on FLARE, G-Nomix, Loter, Recomb-Mix, RFMix, and SALAI-Net. Markers were filtered with minor allele frequency ≤ 0.005 and minor allele count ≤ 50.

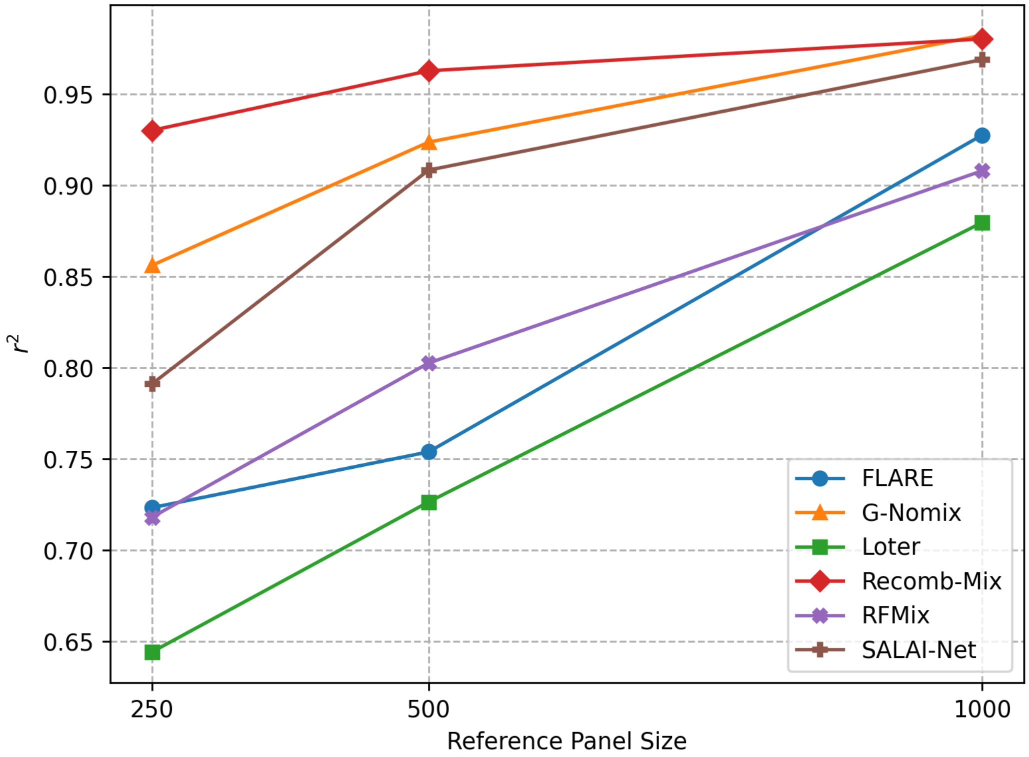

Figure S8: The squared Pearson's correlation coefficient r^2 with the reference panel sizes 250, 500, and 1,000 of the three-way intra-continental simulated datasets on FLARE, G-Nomix, Loter, Recomb-Mix, RFMix, and SALAI-Net. Markers were filtered with minor allele frequency ≤ 0.005 and minor allele count ≤ 50. The reference panel size 100 case was not included because the number of markers was too small and may have influenced the outcome after the filtering.

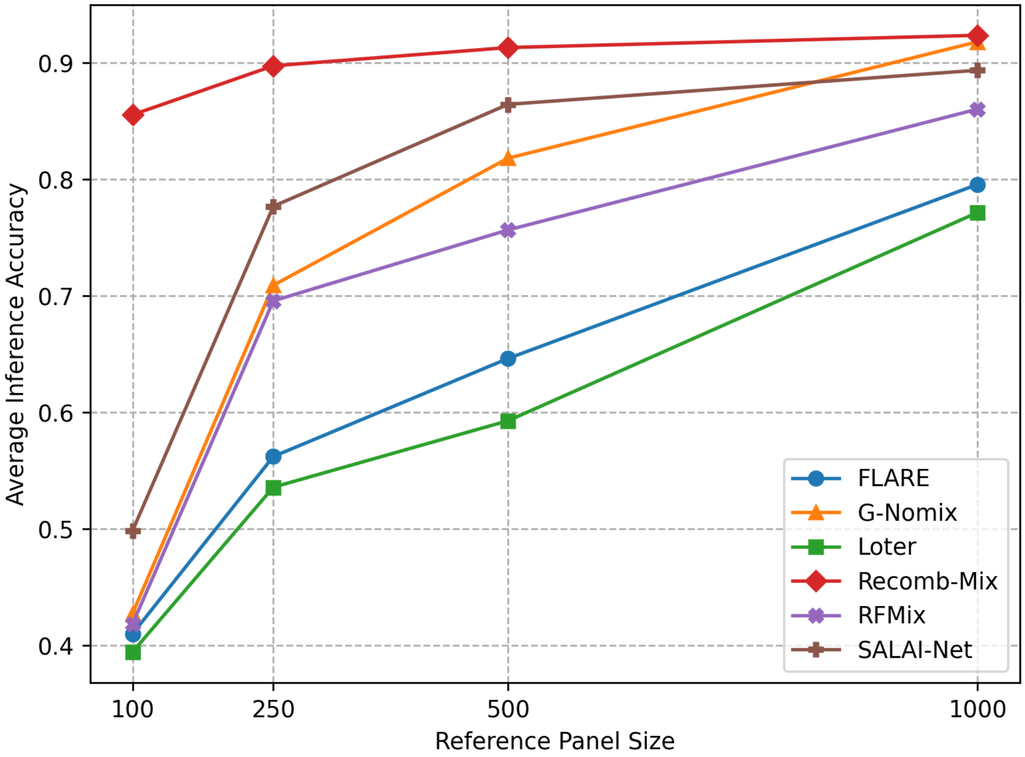

Figure S9: The average accuracy rates with the reference panel sizes 100, 250, 500, and 1,000 of the three-way 15-generation intra-continental simulated datasets on FLARE, G-Nomix, Loter, Recomb-Mix, RFMix, and SALAI-Net.

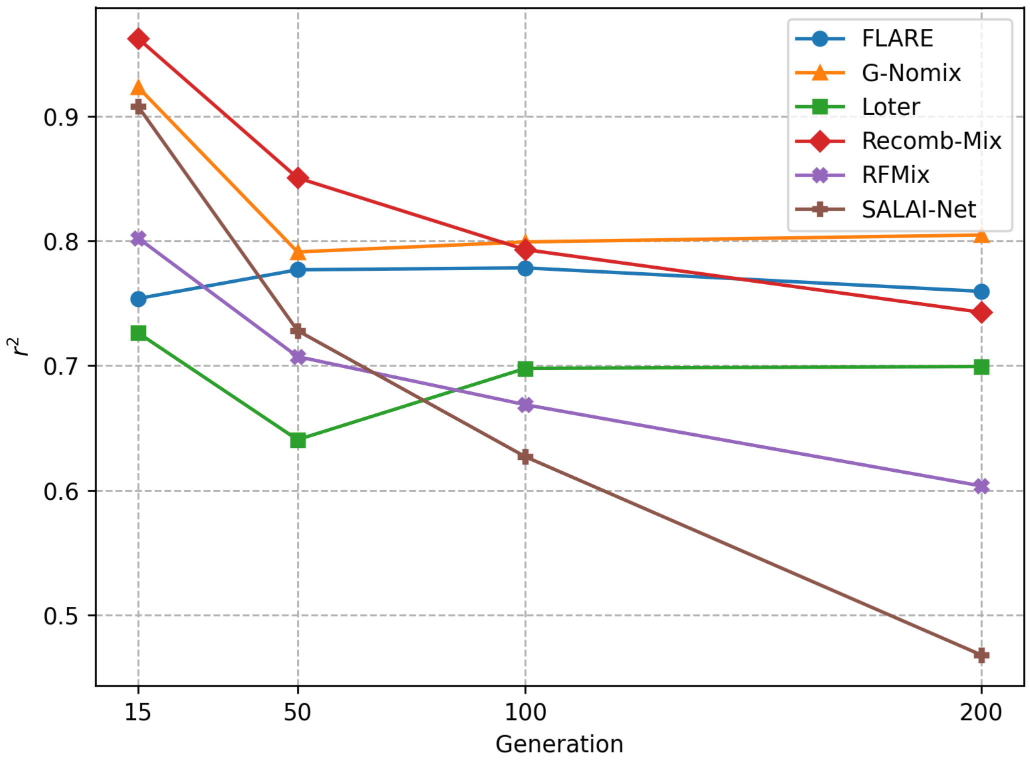

Figure S10: The squared Pearson's correlation coefficient r^2 with the generations 15, 50, 100, and 200 of the three-way intra-continental simulated datasets on FLARE, G-Nomix, Loter, Recomb-Mix, RFMix, and SALAI-Net. Markers were filtered with minor allele frequency ≤ 0.005 and minor allele count ≤ 50.

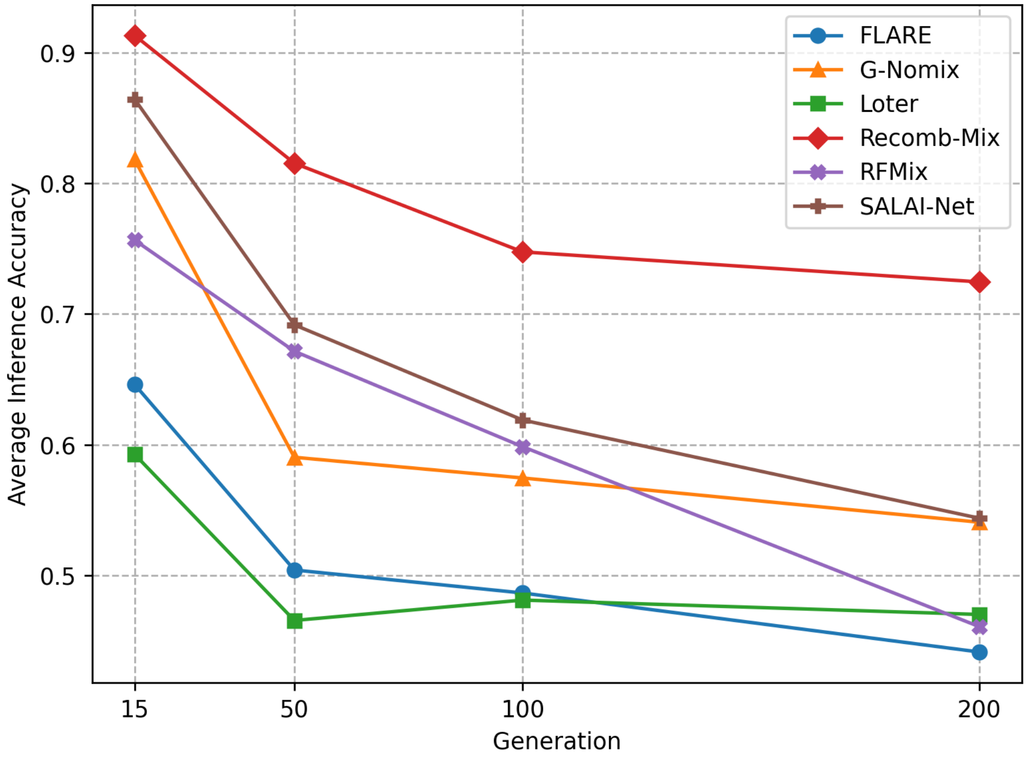

Figure S11: The average accuracy rates with the generations 15, 50, 100, and 200 of the three-way 500-reference intra-continental simulated datasets on FLARE, G-Nomix, Loter, Recomb-Mix, RFMix, and SALAI-Net.

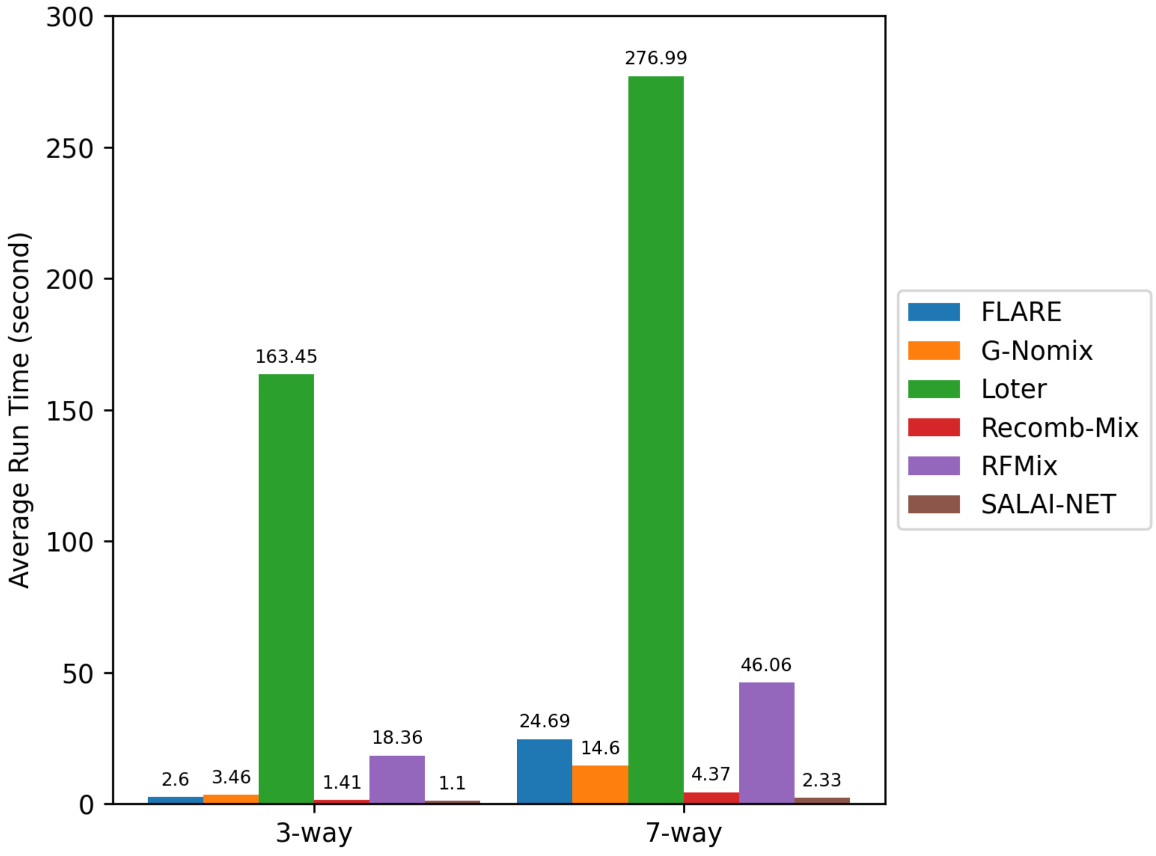

Figure S12: The average run time (second) of LAI methods FLARE, G-Nomix, Loter, Recomb-Mix, RFMix, and SALAI-Net for querying local ancestry information of an admixed individual haplotype on three-way and seven-way reference panels.

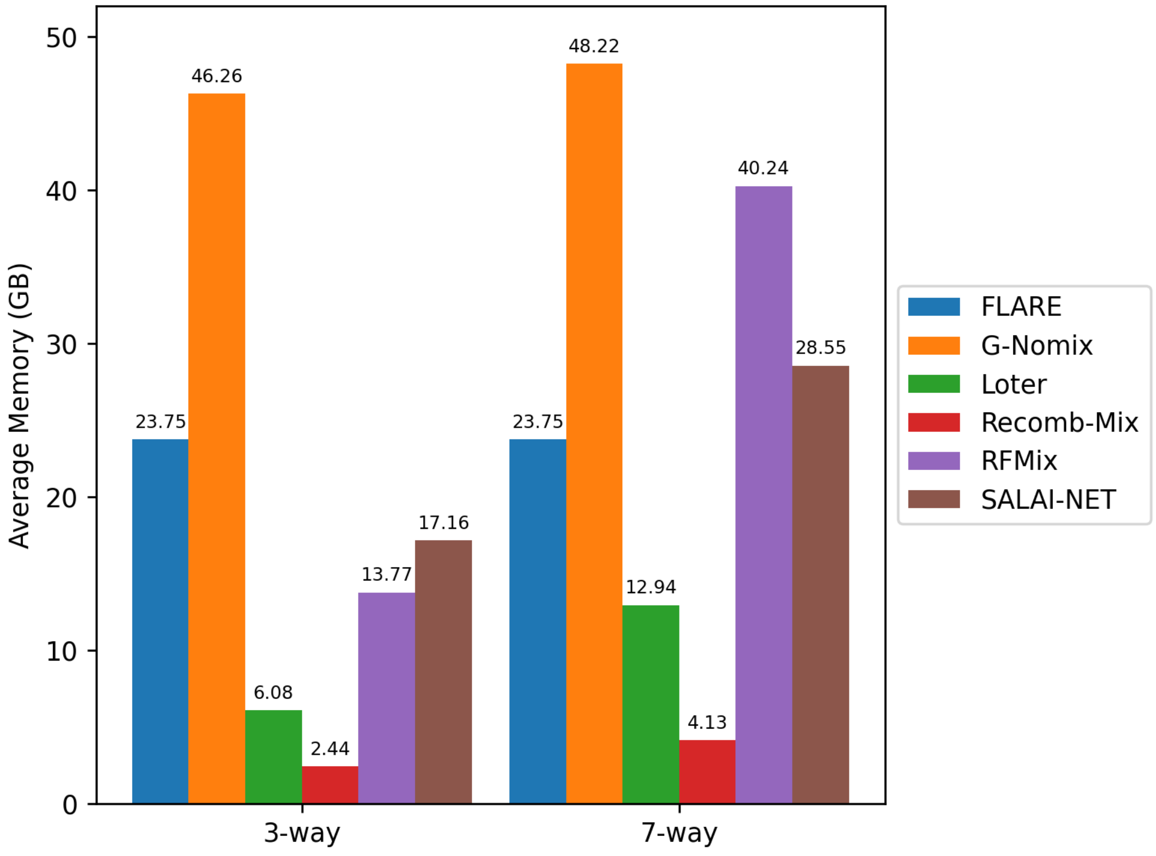

Figure S13: The average memory (GB) of LAI methods FLARE, G-Nomix, Loter, Recomb-Mix, RFMix, and SALAI-Net for querying local ancestry information of an admixed individual haplotype on three-way and seven-way reference panels.

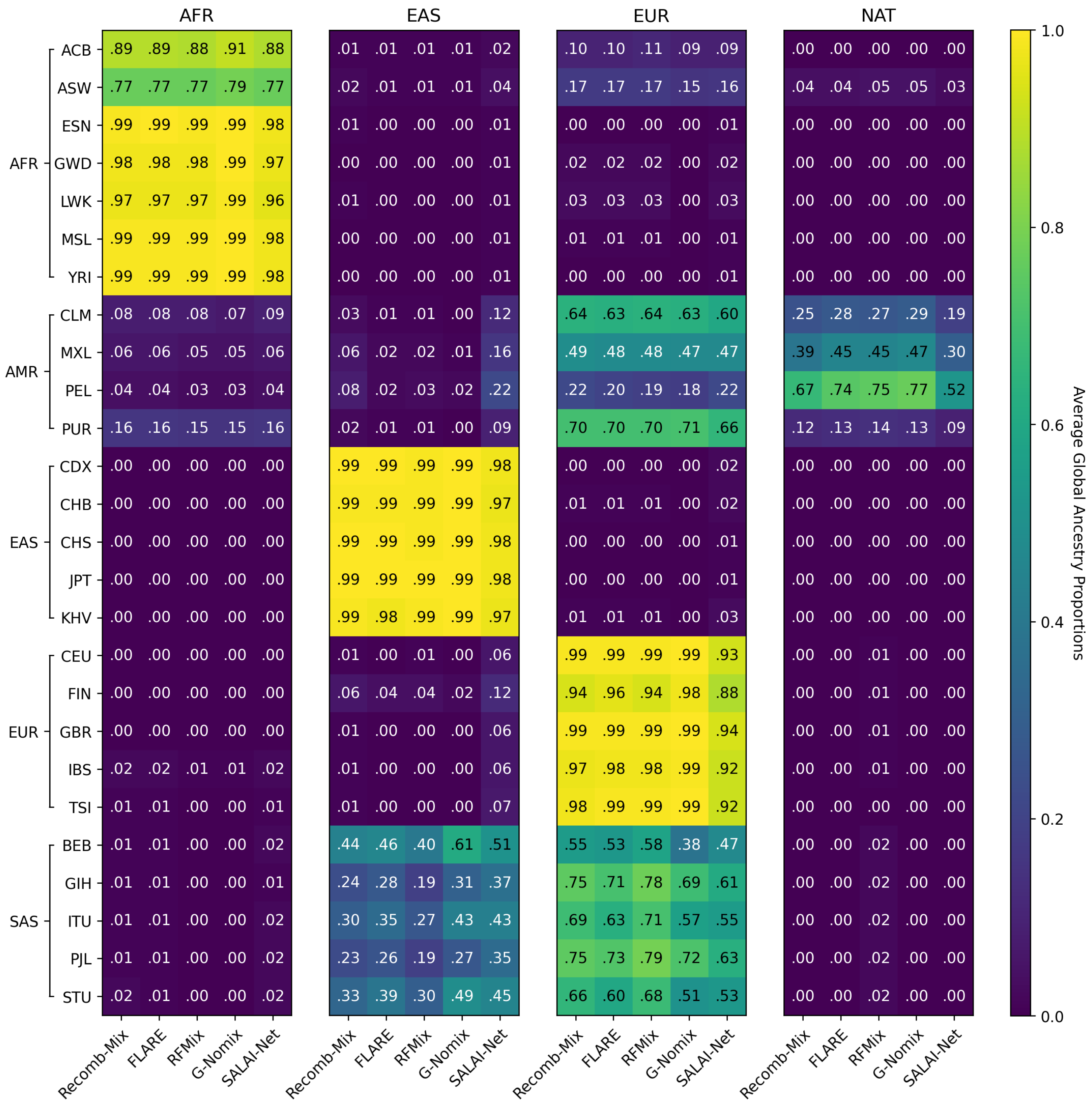

Figure S14: The average global ancestry proportions in the 1000 Genomes Project (TGP) Chromosome 18 data using four reference ancestries from the Human Genome Diversity Project (HGDP) data. The methods are Recomb-Mix, FLARE, RFMix, G-Nomix, and SALAI-Net. Descriptions of the populations are in Supplemental Table S1.

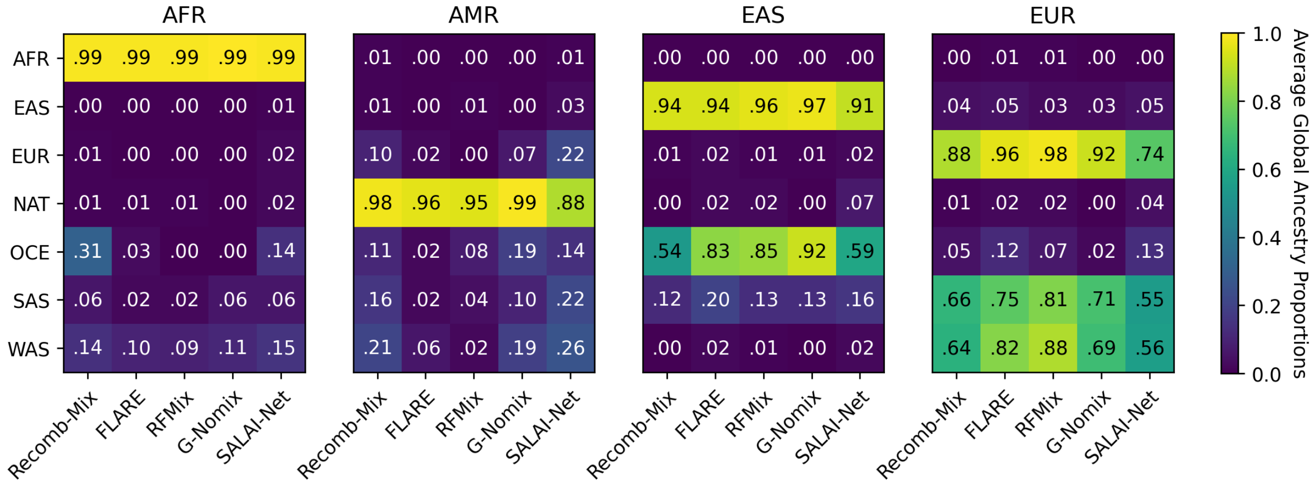

Figure S15: The average global ancestry proportions in the Human Genome Diversity Project (HGDP) Chromosome 18 data using four reference ancestries from the 1000 Genomes Project (TGP) data. The methods are Recomb-Mix, FLARE, RFMix, G-Nomix, and SALAI-Net. Descriptions of the populations are in Supplemental Table S1.

S4 Supplementary Results

S4.1 Availability and usability of discrete ancestry informative markers

The discrete ancestry informative markers (dAIMs) are sufficient to make local ancestry inference calls as they share strong population-specific signals and are densely available on a typical panel. The dAIM densities (i.e., percentages of markers in the dataset being dAIMs) of Chromosome 18 in the HGDP dataset [1]) shown in Figure S16 indicates that dAIMs are evenly distributed across the chromosome, and they share various ancestry signal strengths between populations. There is a dAIM density peak occurring around the 18q21 region in the HGDP dataset (see Figure S16). In a previous admixture mapping study, a genome-wide significant admixture mapping peak contributed from multiple ancestry signals was identified in the same region [3]. This correlation suggests that dAIM density has the potential to identify ancestry-specific selection. Therefore, dAIMs are capable of representing ancestry signals in the panel and are suitable for ancestry inferences.

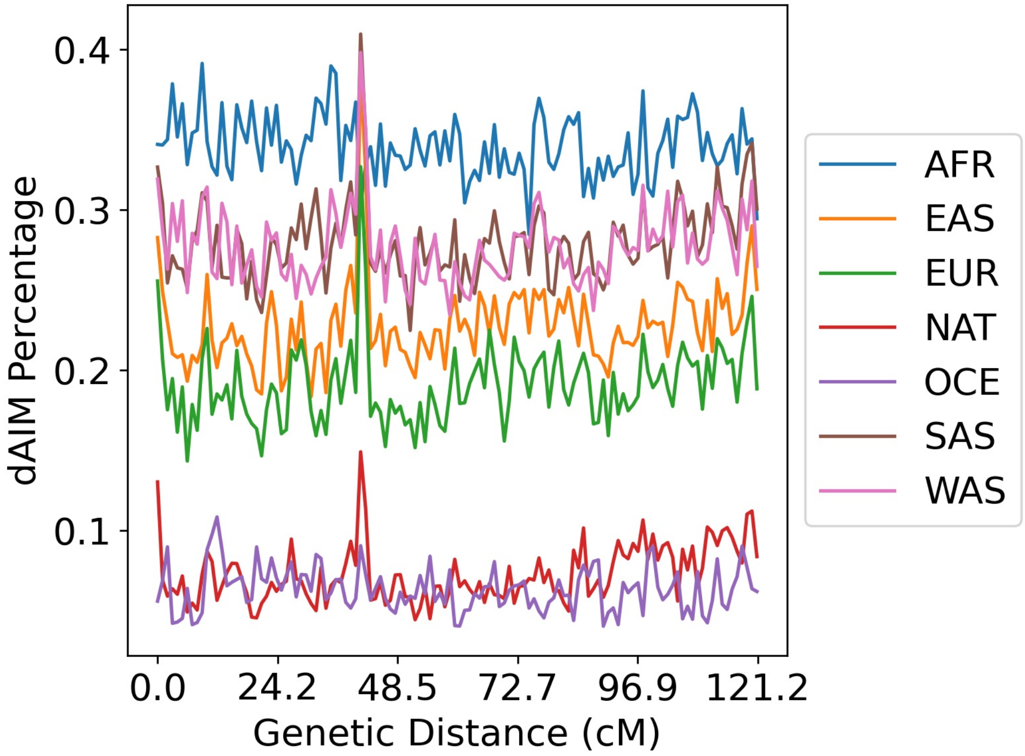

Figure S16: The discrete AIM (dAIM) density in the HGDP dataset per population on Chromosome 18. Each bin is 1 centiMorgan (cM), showing the markers' dAIM percentage.

Since dAIMs share various ancestral population signal strengths across populations (see Figure S16), we would like to know if population signal strength would impact Recomb-Mix's inference accuracy of that population. The squared Pearson's correlation coefficients per population on simulated datasets with various generations were reported in Table S18. The results indicate that populations' r^2 values differ across populations; however, there

Generation	Average	AFR	EAS	EUR	NAT	OCE	SAS	WAS
15	0.9798	0.9999	0.9906	0.9578	0.9961	0.9963	0.9624	0.9552
50	0.9268	0.9979	0.9099	0.8838	0.9726	0.9764	0.8803	0.8669
100	0.8728	0.9597	0.8064	0.8434	0.9154	0.9078	0.8657	0.8111
200	0.5553	0.7356	0.4573	0.6821	0.2995	0.6834	0.3625	0.6670

Table S18: Recomb-Mix's squared Pearson's correlation coefficient r^2 with the generations 15, 50, 100, and 200 of the seven-way 500-reference inter-continental simulated datasets on average and per population. Markers were filtered with minor allele frequency ≤ 0.005 and minor allele count ≤ 50.

is no strong correlation between population signal strengths and associated r^2 values. The population with the strongest ancestry signal (AFR) had the highest r^2 values in all four cases (as shown in Table S18). However, the two populations with the weakest ancestry signals (NAT and OCE) were not the ones with the lowest or the second lowest r^2 values in three out of the four cases.

S4.2 Robustness against admixture with uneven proportions of founders and references

To verify the robustness of Recomb-Mix handling cases on uneven founder populations and reference panels, LAI was experimented with using uneven founders for the imbalanced admixture simulation and uneven reference panels for the inference. Being uneven means the group consists of one-third of individuals from the first population, one-sixth from the second population, and half of individuals from the third population. Being even means the numbers of individuals from the populations in the group are divided equally. Three sets of experiments were performed. One three-way admixture dataset was simulated using even founders and inferred using even references, another was simulated using uneven founders and inferred using even references, and the other was simulated using even founders but inferred using uneven references.

The r^2 values and the accuracy rates in Table S19 and Table S20 indicate that admixed individuals with uneven founders and uneven reference panel slightly impact the performance across all LAI methods. Among all LAI methods, Recomb-Mix had the highest r^2 values and accuracy rates in both cases (0.9426 or 89.20% and 0.8944 or 83.61%, respectively). The process of Recomb-Mix generating a collapsed graph helps convert the unbalanced reference populations into balanced ones. Thus, Recomb-Mix keeps a high accuracy of inference results on the unbalanced reference populations.

Method	Even Founders and References	Uneven Founders	Uneven References
Loter	0.7264	0.7263	0.7097
FLARE	0.7538	0.7187	0.7773
RFMix	0.8024	0.8112	0.7887
SALAI-Net	0.9081	0.8747	0.8692
G-Nomix	0.9235	0.9022	0.8811
Recomb-Mix	**0.9625**	**0.9426**	**0.8944**

Table S19: The squared Pearson's correlation coefficient r^2 of FLARE, G-Nomix, Loter, Recomb-Mix, RFMix, and SALAI-Net performing LAI on three-way 15-generation 500-reference intra-continental simulated datasets with even or uneven number of individuals per population in founder or reference panel. Markers were filtered with minor allele frequency ≤ 0.005 and minor allele count ≤ 50.

Method	Even Founders and References	Uneven Founders	Uneven References
Loter	59.28	51.67	52.98
FLARE	64.62	59.60	56.85
RFMix	75.66	74.61	72.47
G-Nomix	81.83	80.16	73.88
SALAI-Net	86.45	83.07	79.49
Recomb-Mix	**91.33**	**89.20**	**83.61**

Table S20: The average accuracy rates of FLARE, G-Nomix, Loter, Recomb-Mix, RFMix, and SALAI-Net performing LAI on three-way 15-generation 500-reference intra-continental simulated datasets with even or uneven number of individuals per population in founder or reference panel.

Additionally, Recomb-Mix was tested on a modern Latino population admixture model that involves uneven founders, which is a popular realistic model used as a study case for the local ancestry inference [5, 7]. We used SLiM v4.0 to simulate the modern Latino population dataset on Chromosome 1, using the same settings from the RFMix paper [5]. Ten Latino genomes with 45% Native American (NAT), 50% European (CEU), and 5% African ancestry (YRI) were simulated, originating from 400 individuals and 12 generations after the admixture event. 30 individuals from each population were used to form the reference panel. We used Beagle 5.4 to phase the source data. The average LAI accuracy rate using Recomb-Mix and RFMix was 99.36% and 93.79%, respectively. This shows that Recomb-Mix excels in the ancestry inference on the modern Latino population admixture model derived from the uneven founders.

S4.3 Robustness against ancestry misspecification panel

When performing real data analysis, the concern of data imperfection may be raised. Some populations may be less studied and underrepresented in available reference panels. Furthermore, the existing reference populations may contain a small fraction of admixture which may not make them the ideal proxies for the labeled populations. Thus, it is necessary to investigate the impact of the ancestry population misspecification on LAI.

An experiment was conducted by replacing the African reference population in a three-way inter-continental admixed dataset with an imperfect reference panel. The imperfect version of the African reference panel contains individuals who were Africans mixed with Europeans five generations from the start of the simulation. This approach has similar effects as the one MOSAIC had, where their imperfect reference panel contained admixed Sub-Saharan Africans and Europeans [6]. We did not follow their process because the sampled individuals they used for the simulation were from the extended HGDP dataset, whose data density is only at the single nucleotide polymorphism (SNP) array level [4]. The ancestry misspecification experiments were repeated for 15, 50, 100, and 200 generations since the admixture event, and FLARE, G-Nomix, Loter, Recomb-Mix, RFMix, and SALAI-Net were tested. We did not include MOSAIC as it was designed for the case when the source population lacked the availability of WGS data [6].

All LAI methods were impacted by the misspecification reference panel but still performed well, as shown in Figures S17 and S18 (values are in Tables S3 and S4). Under the r^2 criteria with markers having minor allele values filtered, Recomb-Mix performed the best in the cases of 50 and 100 generations since the admixture event. RFMix performed the best for the most recent admixture case, and FLARE performed the best for the most distant admixture case. Without filtering out any markers, Recomb-Mix had the highest accuracy rate for most cases except the 15-generation case where RFMix performed the best.

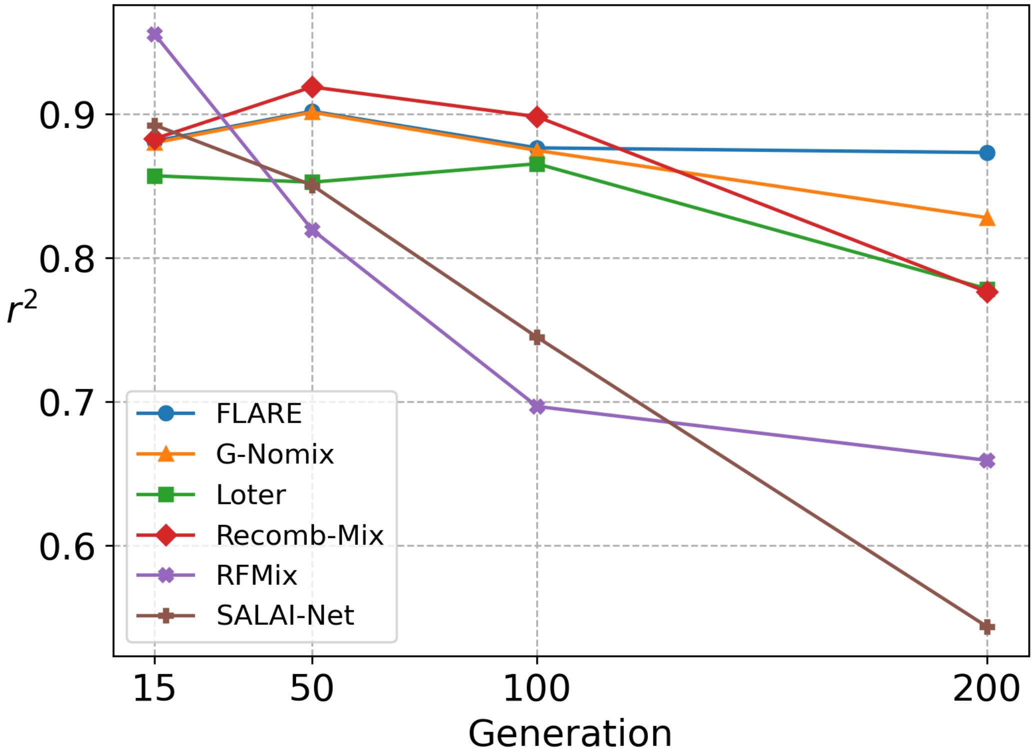

Figure S17: The squared Pearson's correlation coefficient r^2 of local ancestry inference with generations 15, 50, 100, and 200 of the three-way 500-misspecified-reference inter-continental simulated datasets on FLARE, G-Nomix, Loter, Recomb-Mix, RFMix, and SALAI-Net. Markers were filtered with minor allele frequency ≤ 0.005 and minor allele count ≤ 50.

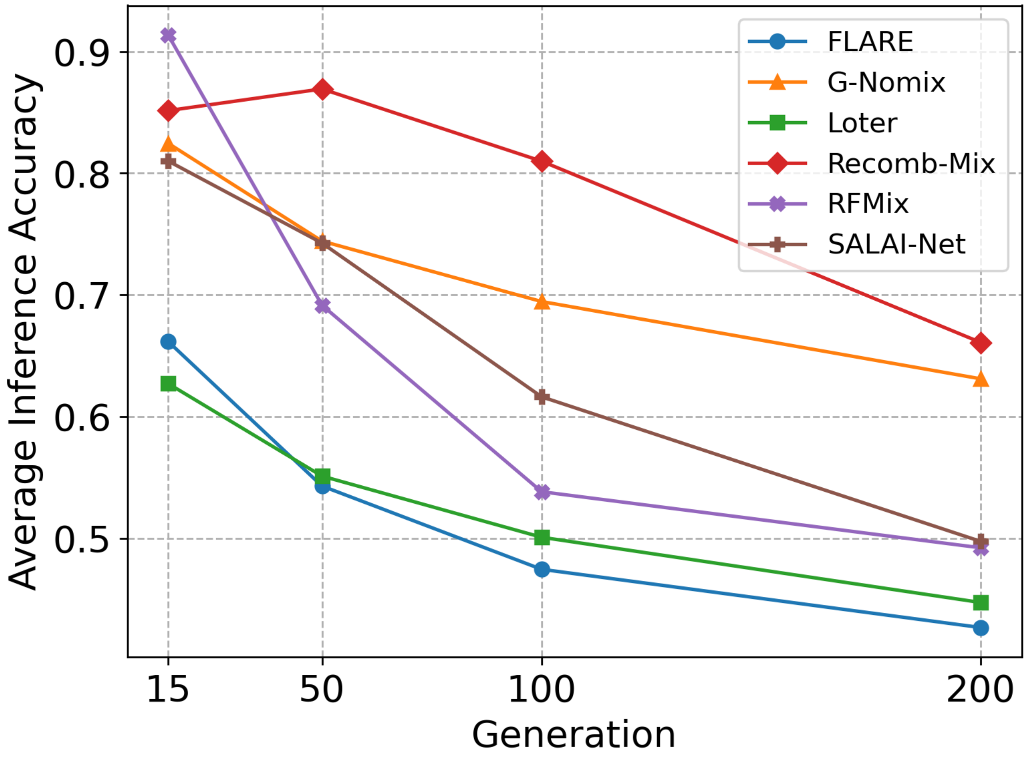

Figure S18: The average accuracy rates of local ancestry inference with generations 15, 50, 100, and 200 of the three-way 500-misspecified-reference inter-continental simulated datasets on FLARE, G-Nomix, Loter, Recomb-Mix, RFMix, and SALAI-Net.

**Method**	**15**	**50**	**100**	**200**
FLARE	0.8811	0.9019	0.8764	0.8731
G-Nomix	0.8799	0.9012	0.8746	0.8278
Loter	0.8570	0.8525	0.8653	0.7784
Recomb-Mix	0.8827	0.9188	0.8981	0.7762
RFMix	0.9556	0.8195	0.6965	0.6590
SALAI-Net	0.8923	0.8504	0.7447	0.5433

Table S21: The squared Pearson's correlation coefficient r^2 with the generations 15, 50, 100, and 200 of the three-way 500-misspecified-reference inter-continental simulated datasets on FLARE, G-Nomix, Loter, Recomb-Mix, RFMix, and SALAI-Net. Markers were filtered with minor allele frequency ≤ 0.005 and minor allele count ≤ 50.

Method	15	50	100	200
FLARE	66.16	54.29	47.44	42.65
G-Nomix	82.46	74.42	69.45	63.10
Loter	62.71	55.11	50.08	44.71
Recomb-Mix	85.15	86.93	80.99	66.08
RFMix	91.36	69.15	53.82	49.21
SALAI-Net	81.00	74.26	61.62	49.73

Table S22: The average accuracy rates with the generations 15, 50, 100, and 200 of the three-way 500-misspecified-reference inter-continental simulated datasets on FLARE, G-Nomix, Loter, Recomb-Mix, RFMix, and SALAI-Net.

S4.4 Robustness against phasing error

To investigate the impact of phasing error on local ancestry inference, two cases of phasing errors on either the target panel or the reference panel were explored. The three-way 15-generation 100-reference inter-continental simulated dataset was used as the baseline, and Beagle 5.4 was applied to phase the panels. After completing the phasing process, the phasing error rates were measured for both panels. The results showed a phasing error rate of 0.58% for the target panel and 1.33% for the reference panel. FLARE, G-Nomix, Loter, Recomb-Mix, RFMix, and SALAI-Net were tested on the panels and diploid accuracy rates were used for the performance measurement as RFMix did [5]. The results in Table S23 show that the diploid accuracy rates did not fluctuate much when there were phasing errors on the panels, indicating that the low rate of phasing errors may not have a substantial impact on the local ancestry inference.

Method	No Phasing Error	Phasing Error on Targets	Phasing Error on References
Loter	64.00	62.72	63.35
FLARE	63.68	63.61	64.15
RFMix	72.52	71.26	72.77
SALAI-Net	86.81	85.82	87.54
G-Nomix	86.70	86.37	86.54
Recomb-Mix	97.97	97.39	97.98

Table S23: The diploid accuracy rates of FLARE, G-Nomix, Loter, Recomb-Mix, RFMix, and SALAI-Net performing LAI on three-way 15-generation 100-reference inter-continental simulated datasets with no phasing error, phasing error on target panel, and phasing error on reference panel.

S4.5 Ablation study

We want to understand which component contributes the most to Recomb-Mix's ancestry inference process. Experiments were designed for Recomb-Mix to make inferences on a three-way inter-continental 15-generation 100-reference simulated dataset by not setting the within-population template change penalty to zero or not using the recombination rates. We observed a slight decrease in performance when the recombination rate was not used. However, the performance dropped significantly when the within-population template change penalty was applied (see Figure S19 and Table S24). Figure S19 shows LAI accuracies are significantly improved when setting the within-population template change penalty to zero, especially for small reference panel cases. We also calculated the average number of threading path changes across populations and the standard deviations of the dataset with $228,503$ markers for Recomb-Mix and the version that used the within-population template change penalty. Recomb-Mix had 8.47 ± 2.54, while the latter version had a much larger number, 198.46 ± 46.94. The LAI calling became less effective when using the template change penalty within each population. This may be due to the numerous local optimal threading paths to be explored within each population, which can lead to noise and deviation from finding the path with the minimal global penalty score. By setting the within-population template change penalty to zero, the number of explorations between the paths within a population is significantly reduced, and the focus is shifted to only a few consolidated paths representing diverse haplotype templates.

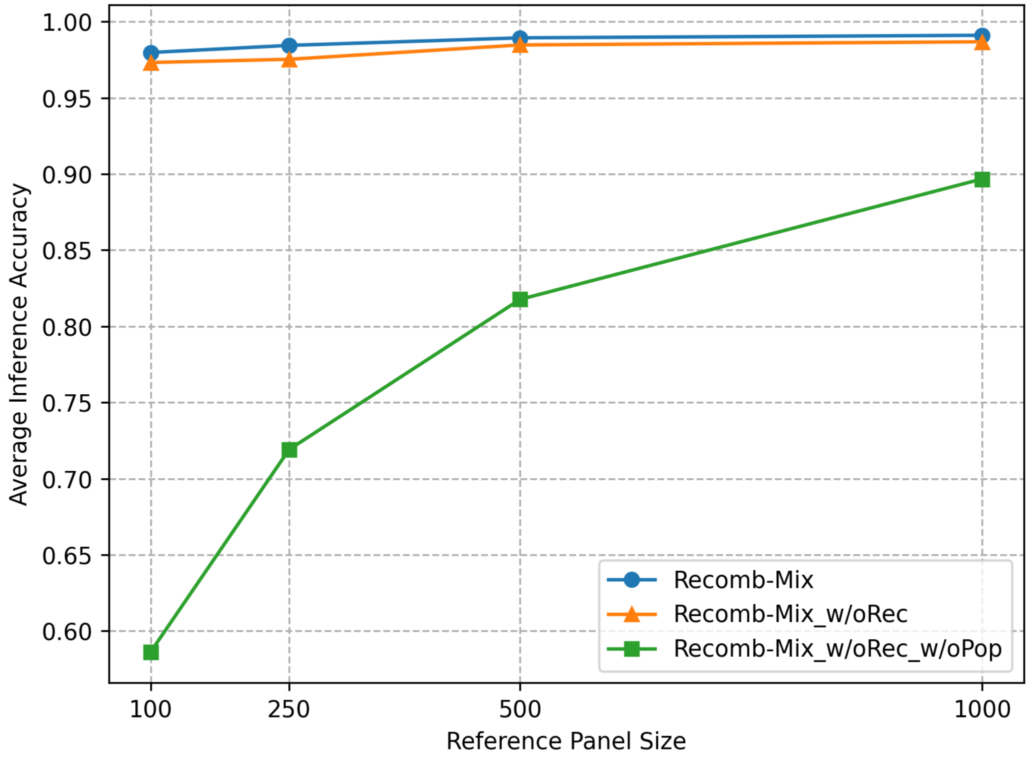

Figure S19: The average accuracy rates with reference panel sizes 100, 250, 500, and 1,000 of the three-way 15-generation inter-continental simulated datasets. Recomb-Mix_w/oRec method is Recomb-Mix without using recombination rates in the objective function. Instead, a constant value is used as the template change penalty. Recomb-Mix_w/oRec_w/oPop is Recomb-Mix without using recombination rates in the objective function and without considering the zero template change penalty within each population. Instead, the template change penalty occurs when the threading path is changed to a different haplotype template from the current one, regardless of their population labels, as Loter did.

**Method**	**100**	**250**	**500**	**1,000**
Recomb-Mix	97.97	98.44	98.93	99.10
Recomb-Mix_w/oRec	97.32	97.53	98.47	98.68
Recomb-Mix_w/oRec_w/oPop	58.62	71.91	81.77	89.67

Table S24: The average accuracy rates of LAI methods Recomb-Mix, Recomb-Mix_w/oRec, and Recomb-Mix_w/oRec_w/oPop on the three-way 15-generation inter-continental simulated datasets with reference panel sizes 100, 250, 500, and 1,000.

The dAIMs are usually evenly distributed alongside the chromosome, as we showed in Figure S16. To illustrate dAIM's important role in LAI, we designed an experiment to engineer a new dataset based on the simulated one

by taking out all the dAIMs for certain regions. We tested Recomb-Mix on the engineered dataset, and the result showed a strong correlation between the dAIM density and the accuracy of the inference. Figures S20 and S21 show the dAIM density and local ancestry inference accuracy rate of the original dataset and the engineered dataset. In Figure S21, there are five instances where the low LAI accurate rates correspond with areas lacking dAIMs in the engineered dataset. The Pearson correlation coefficient for this dataset's dAIM density and local ancestry inference accuracy rate is 0.79, demonstrating a strong correlation between dAIMs and LAI accuracies.

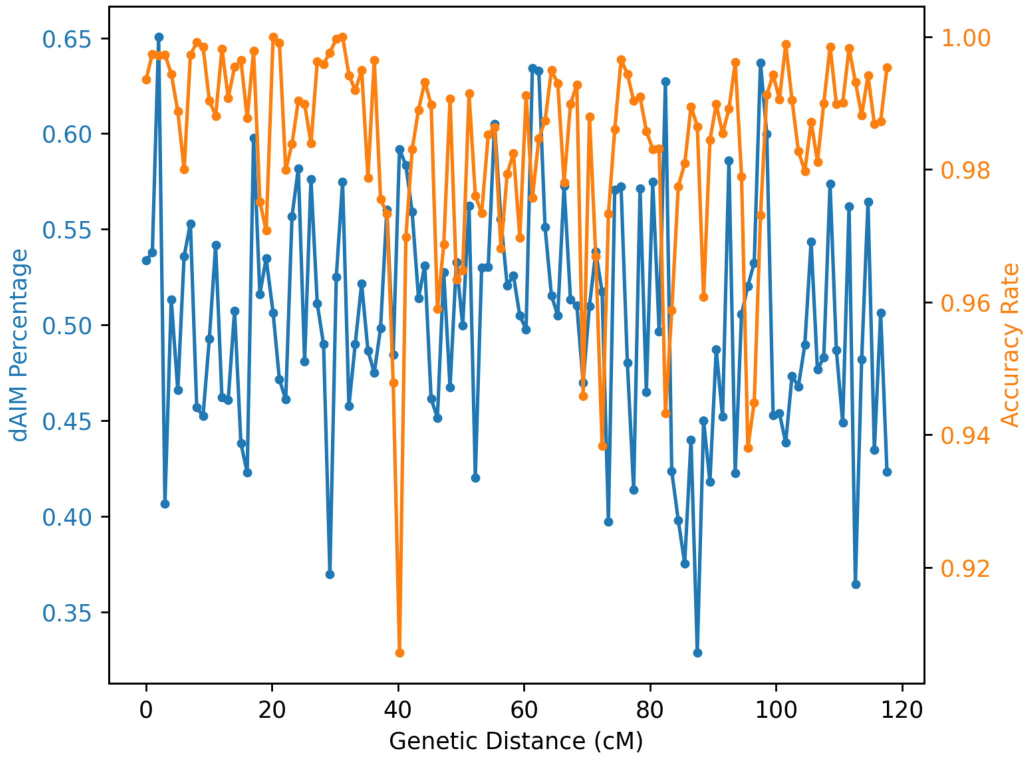

Figure S20: The discrete AIM (dAIM) density and local ancestry inference accuracy rate of a three-way 15-generation 100-reference inter-continental Chromosome 18 simulated dataset. Each bin is 1 centiMorgan (cM), showing the markers' dAIM percentage and the average accuracy rate of local ancestry inferred by Recomb-Mix.

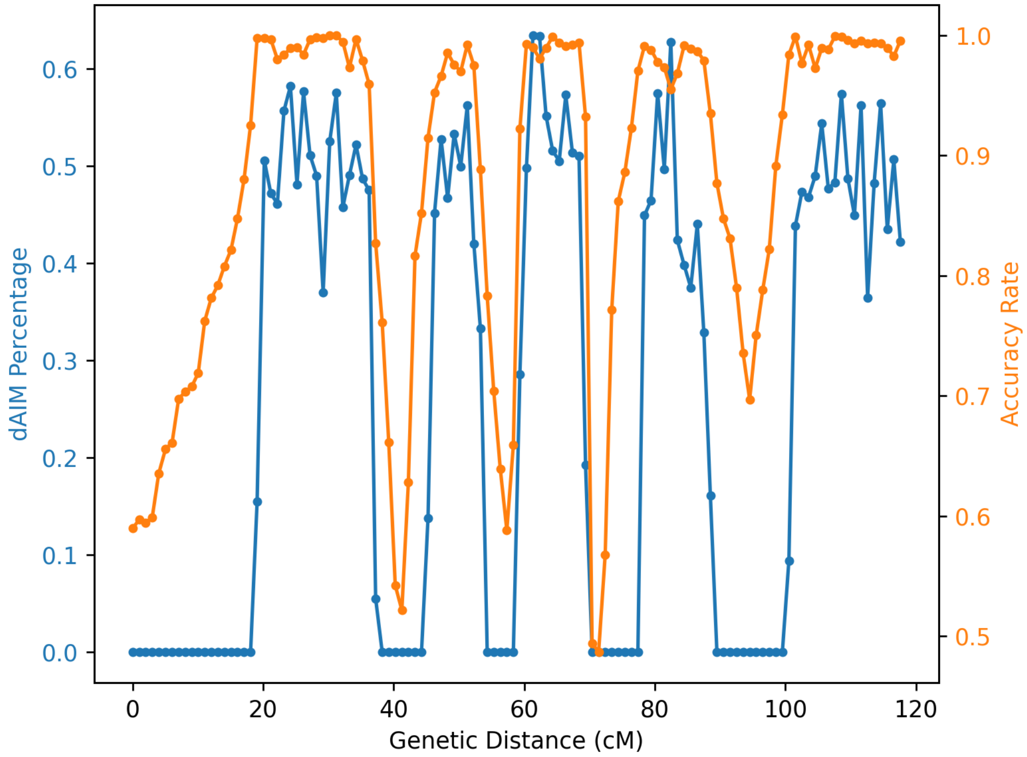

Figure S21: The discrete AIM (dAIM) density and local ancestry inference accuracy rate of a three-way 15-generation 100-reference inter-continental Chromosome 18 simulated dataset. The dataset was engineered to have certain regions of dAIMs removed. Each bin is 1 centiMorgan (cM), showing the markers' dAIM percentage and the average accuracy rate of local ancestry inferred by Recomb-Mix.

Recomb-Mix's performance may be impacted when the panel has a limited number of dAIMs (i.e., the panel has most non-dAIM markers which have a minor allele frequency in the range (0, 0.5]). This situation may occur if the reference panel consists of a very large number of individuals or if the panel includes common variants only. In such cases, the compact population graph may lack strong ancestry signals to perform LAI.

To address this challenging situation when the reference panel has very few or no dAIMs presented, Recomb-Mix offers an option to filter out certain allele values based on their minor allele frequencies. This filtering process increases the number of dAIMs in the panel, allowing Recomb-Mix to perform LAI efficiently; however, it comes at the cost of a resolution-reduced panel. The low-resolution reference panel could impact the LAI accuracy as some of the ancestry information may be lost compared to the original panel.

We tested the filtering option on an engineered dataset to have all dAIMs removed from a simulated Chromosome 18 panel having 200 admixed individuals with 500 individuals in the reference panel. The original dataset was simulated by msprime using the American admixture model [2]. Then, we filtered out markers whose minor allele frequency is ≤ 0.01, yielding an engineered dataset that has no dAIMs. The number of markers was reduced from 1,103,446 to 109,240. Recomb-Mix was tested on the original dataset, the engineered dataset, and the engineered dataset using the filtering option with threshold values set to 0.05 and 0.1. Table S25 presents the dAIM percentages

and average accuracy rates from our experiment. Recomb-Mix achieved a 99% accuracy rate on the original dataset, while the accuracy dropped to about 15% on an engineered dataset that did not include any dAIMs. When we applied the filtering option, the dAIM percentage became non-zero and the accuracy rate improved significantly (up to 87%). However, we observed that increasing the filtering threshold led to a decrease in panel resolution, which negatively impacted the accuracy rate (down to 79%). This suggests that Recomb-Mix may underperform on a compact population graph with no dAIMs, as it provides very limited ancestry information. Utilizing the filtering option with a small threshold value would substantially improve the performance of Recomb-Mix for the case when there are no dAIMs in the panel.

	**Original**	**Engineered**	**Engineered (threshold=0.05)**	**Engineered (threshold=0.1)**
dAIM Percentage	0.23	0.00	0.01	0.04
Accuracy Rate	99.96	14.93	87.02	79.87

Table S25: The dAIM percentages and average accuracy rates of Recomb-Mix on the three-way 500-reference American admixture simulated original dataset and engineered datasets using filtering threshold 0, 0.05, and 0.1.

References

[1] Anders Bergström, Shane A. McCarthy, Ruoyun Hui, Mohamed A. Almarri, Qasim Ayub, Petr Danecek, Yuan Chen, Sabine Felkel, Pille Hallast, Jack Kamm, et al. Insights into human genetic variation and population history from 929 diverse genomes. *Science*, 367(6484):eaay5012, 2020.

[2] Sharon R. Browning, Brian L. Browning, Martha L. Daviglus, Ramon A. Durazo-Arvizu, Neil Schneiderman, Robert C. Kaplan, and Cathy C. Laurie. Ancestry-specific recent effective population size in the americas. *PLOS Genetics*, 14(5):1–22, 05 2018.

[3] Christopher R. Gignoux, Dara G. Torgerson, Maria Pino-Yanes, Lawrence H. Uricchio, Joshua Galanter, Lindsey A. Roth, Celeste Eng, Donglei Hu, Elizabeth A. Nguyen, Scott Huntsman, et al. An admixture mapping meta-analysis implicates genetic variation at 18q21 with asthma susceptibility in Latinos. *Journal of Allergy and Clinical Immunology*, 143(3):957–969, 2019.

[4] Garrett Hellenthal, George B. J. Busby, Gavin Band, James F. Wilson, Cristian Capelli, Daniel Falush, and Simon Myers. A genetic atlas of human admixture history. *Science*, 343(6172):747–751, 2014.

[5] Brian K. Maples, Simon Gravel, Eimear E. Kenny, and Carlos D. Bustamante. RFMix: A discriminative modeling approach for rapid and robust local-ancestry inference. *The American Journal of Human Genetics*, 93(2):278–288, Aug 2013.

[6] Michael Salter-Townshend and Simon Myers. Fine-scale inference of ancestry segments without prior knowledge of admixing groups. *Genetics*, 212(3):869–889, 05 2019.

[7] Yong Wang, Shiya Song, Joshua G. Schraiber, Alisa Sedghifar, Jake K. Byrnes, David A. Turissini, Eurie L. Hong, Catherine A. Ball, and Keith Noto. Ancestry inference using reference labeled clusters of haplotypes. *BMC Bioinformatics*, 22(1):459, Sep 2021.
